# Molecular detection and identification of Diatrypaceous airborne spores in Australian vineyards revealed high species diversity between regions

**DOI:** 10.1371/journal.pone.0286738

**Published:** 2023-06-02

**Authors:** Regina Billones-Baaijens, Meifang Liu, Mark R. Sosnowski, Matthew R. Ayres, Sandra Savocchia

**Affiliations:** 1 Gulbali Institute, Charles Sturt University, Wagga Wagga, NSW, Australia; 2 School of Agricultural, Environmental and Veterinary Sciences, Charles Sturt University, Wagga Wagga, NSW, Australia; 3 South Australian Research and Development Institute, Adelaide, SA, Australia; 4 School of Agriculture, Food and Wine, Waite Research Institute, The University of Adelaide, Adelaide, SA, Australia; Universidade do Minho, PORTUGAL

## Abstract

The grapevine trunk disease, Eutypa dieback (ED), causes significant vine decline and yield reduction. For many years, the fungus *Eutypa lata* was considered the main pathogen causing ED of grapevines in Australia. Recent studies showed other Diatrypaceous fungi were also associated with vines exhibiting dieback symptoms but there is limited information on how these fungal pathogens spread in vineyards. Thus, information on the spore dispersal patterns of Diatrypaceous fungi in different wine regions will assist in identifying high-risk infection periods in vineyards. Using more than 6800 DNA samples from airborne spores collected from eight wine regions in south-eastern Australia over 8 years using a Burkard spore trap, this study investigated the diversity and abundance of Diatrypaceous species, using multi-faceted molecular tools. A multi-target quantitative PCR (qPCR) assay successfully detected and quantified Diatrypaceous spores from 30% of the total samples with spore numbers and frequency of detection varying between regions and years. The high-resolution melting analysis (HRMA) coupled with DNA sequencing identified seven species, with *E*. *lata* being present in seven regions and the most prevalent species in the Adelaide Hills, Barossa Valley and McLaren Vale. *Cryptovalsa ampelina* and *Diatrype stigma* were the predominant species in the Clare Valley and Coonawarra, respectively while *Eutypella citricola* and *Eu*. *microtheca* dominated in the Hunter Valley and the Riverina regions. This study represents the first report of *D*. *stigma* and *Cryptosphaeria multicontinentalis* in Australian vineyards. This study further showed rainfall as a primary factor that triggers spore release, however, other weather factors that may influence the spore release in different climatic regions of Australia still requires further investigation.

## Introduction

**Fig 1:**
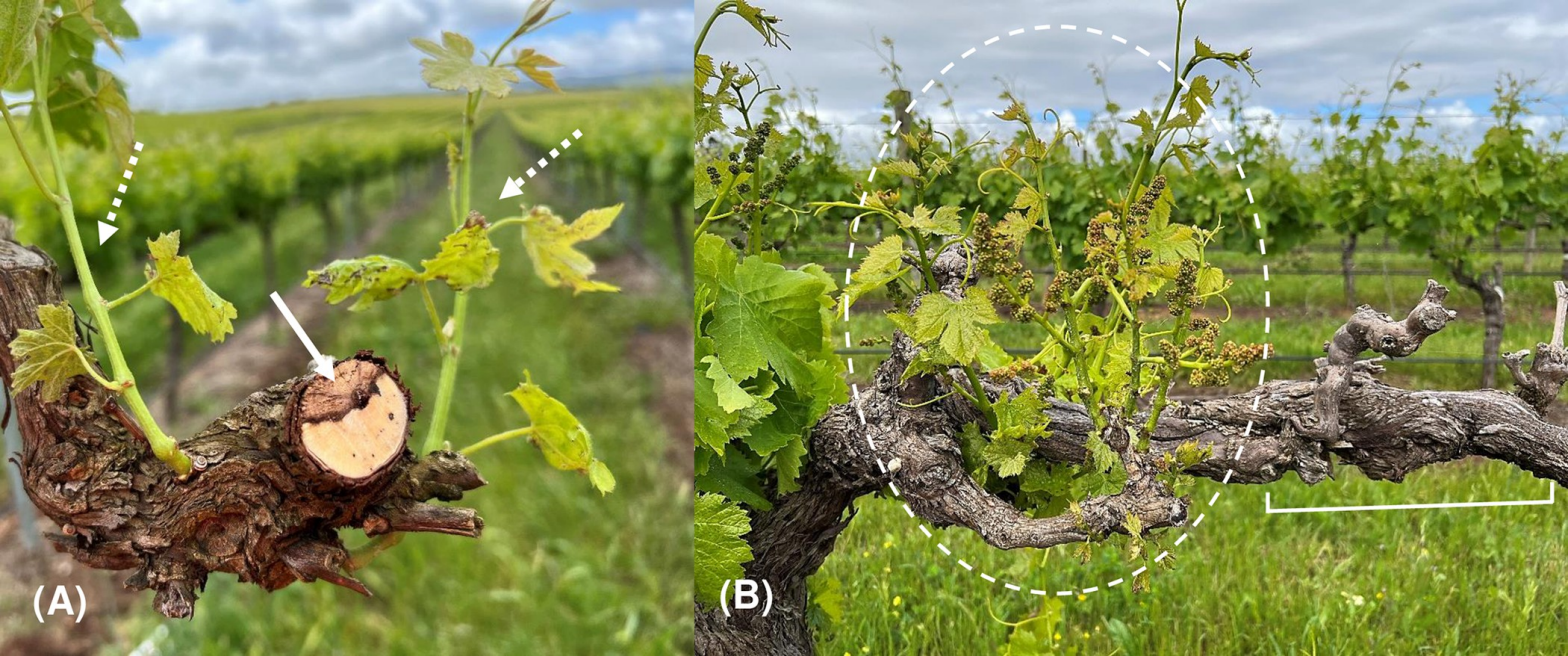
Symptoms of Eutypa dieback of grapevines caused by *Eutypa lata*. (A) Cross-section of the cordon with wedge-shaped wood necrosis (solid arrow) and stunted shoots (dashed arrows); (B) vine cordon with dieback (bracket) and chlorotic, stunted shoots (dashed circle).

Eutypa dieback (ED) is a major trunk disease of grapevines worldwide, causing significant yield reduction and threatening the sustainability of vineyards. The fungus *Eutypa lata* (syn *E*. *armeniaceae*), first described as a dieback pathogen of apricots [1] was also found to be responsible for the dieback and vascular disease of grapevines in Australia [2] and abroad [3, 4]. Infection in vineyards occurs when the ascospores of *E*. *lata* land on fresh pruning wounds, germinate and colonise the xylem vessels of the cordons and trunk. This infection will eventually develop into cankers and a characteristic wedge-shaped necrosis of the wood (Fig 1A). Infected vines develop dieback and foliar symptoms that include stunted shoots with short internodes, and small chlorotic, distorted leaves with necrotic margins (Fig 1B). These foliar symptoms are mainly due to the phytotoxic compounds produced by *E*. *lata* which are translocated into the foliage of the vine during the growing season [5–9]. If not controlled, the fungus eventually kills infected vines. In Australia, ED is widespread in many wine-growing regions and is becoming more prevalent as vineyards age [10–12]. This disease is now considered a serious threat to the sustainability of the Australian wine industry that contributes $45.5 billion to the value of gross output for Australia [13].

For many years, *E*. *lata* was considered the sole pathogen causing ED of grapevines [4, 14] but more recent studies showed other Diatrypaceous species closely related to *E*. *lata* were also associated with the decline and dieback of grapevines [15–18]. The Diatrypaceae family is made up of eight genera with *Cryptosphaeria*, *Cryptovalsa*, *Diatrype*, *Diatrypella*, *Eutypa*, and *Eutypella* being the most common [17]. Australian vineyard surveys in 2008–2009 identified seven species associated with grapevines, namely, *Cryptovalsa ampelina*, *C*. *rabenhorstii*, *Diatrype* spp., *Diatrypella vulgaris*, *E*. *lata*, *Eutypella citricola* and *Eu*. *microtheca* [18]. The same survey further isolated *Cryptosphaeria* spp., *Diatrype brunneospora*, and *Eutypa leptoplaca* from other woody hosts including *Populus* spp., *Acacia* spp., *Ficus* and *Quercus* sp. [18]. These surveys further revealed that *E*. *lata* was the only species associated with vines exhibiting both foliar symptoms and wood canker while other Diatrypaceous species such as *Eutypella* spp. *Diatrypella*, spp. and *C*. *ampelina* were only associated with vines with wood cankers [18–20]. Subsequent pathogenicity studies showed these Diatrypaceous species produced internal necrosis and staining of wood, with *E*. *lata* with being the most virulent species and the only species that produced foliar symptoms [11, 19, 21]. A pathogenicity study in South Africa further showed that the virulence of *C*. *ampelina*, *Eu*. *microtheca* and *Eu*. *citricola* in grapevines was comparable to that of *E*. *lata* [22].

While the spore release patterns of *E*. *lata* have been well studied for many years, limited work has been conducted to investigate the spore dispersal patterns of other Diatrypaceous pathogens in vineyards. The only recent studies that investigated the release of other Diatrypaceous spores were carried out in British Columbia, Canada [23] and in Southern California [24]. Since vineyard surveys in Australia showed that *E*. *lata* was absent in certain wine regions and other Diatrypaceous species were the main pathogens associated with the vine cankers and dieback in some vineyards [18–20, 25], there is a need to investigate the spore release patterns of different Diatrypaceous species. This will assist with the investigations on the climatic conditions required for spore release by Diatrypaceous pathogens and inoculum dispersal throughout the growing season and during the pruning season in different climatic regions of Australia.

Previous studies on the spore dispersal studies for *E*. *lata* relied primarily on conventional techniques such as microscopy and culturing of the microorganisms on artificial media, which are extremely time consuming, and are limited in accuracy and sensitivity in detecting these pathogens from environmental samples [24, 26, 27]. In recent years, PCR-based molecular techniques have been reported to be useful for detecting airborne pathogens from different environmental samples due to their accuracy and sensitivity compared to conventional plant pathology techniques. Quantitative PCR (qPCR) has since been commonly used to detect airborne inoculum of different grapevine pathogens including the bunch rot pathogen *Botrytis cinerea* [28], the powdery mildew pathogen *Erysiphe necator* [29] and the Asian grapevine leaf rust pathogen *Neophysopella tropicalis* [30]. For grapevine trunk disease pathogens, a qPCR assay was developed to amplify multiple species of Botryosphaeria dieback pathogen spores from spore tape samples [31]. A qPCR method was also developed to investigate the temporal dispersal of the Petri disease pathogen, *Phaeomoniella chlamydospora* airborne inoculum in Spanish vineyards [32]. For ED, quantitative PCR assays to detect *E*. *lata* in grapevine wood [33, 34] and spore trap samples [35] were further developed in recent years. However, these qPCR assays are only specific to *E*. *lata*. Since there are at least six other Diatrypaceous species aside from *E*. *lata* that are known to be present in Australian vineyards, a multi-target qPCR assay that can detect all these Diatrypaceous pathogens in one sample will allow rapid and more cost-effective detection and quantification of these airborne pathogen spores.

High resolution melting analysis (HRMA) is a simple PCR-based method that is used for rapid and accurate plant pathogen identification and genotyping [36–38]. This analysis is usually carried out by increasing the temperature at the end of the PCR amplification cycle, and the decrease in fluorescence due to the “melting” of double stranded DNA is then measured and melting profiles for each amplicon are generated. The HRMA melting profiles are dependent on the GC content, length and sequence of the PCR product [39]. Thus, amplicons of different sizes and GC content will potentially generate unique HRMA profiles that will allow identification of different species and genotypes. The HRMA approach has been applied widely for screening pathogenic variants [40], disease associated mutations [36], and species identification and genotyping of plant pathogens [38, 41, 42]. Thus, a multi-target qPCR coupled with a HRMA protocol that can generate specific melting profiles for different Diatrypaceous species can potentially provide a simple alternative method for simultaneous identification of Diatrypaceous species in mixed DNA samples.

This study investigated the diversity of Diatrypaceous airborne spores from different wine growing regions in Australia using multi-faceted DNA-based molecular tools. Molecular tools were developed to detect and quantify Diatrypaceous pathogens associated with ED in Australian vineyards. These molecular tools allowed the detection, identification and quantification Diatrypaceous inoculum from Burkard spore tape samples.

## Materials and methods

### Primer design

To design the multi-target primers, reference sequences that included *Cryptovalsa ampelina* (7), *C*. *rabenhorstii*, *Diatrypella vulgaris* (6), *E*. *lata* (17), *E*. *leptoplaca* (10), (4), *Eutypella citricola* (12), *Eu*. *cryptovalsoidea* (2) and *Eu*. *microtheca* (12) were retrieved from GenBank (http://www.ncbi.nlm.nih.gov/). All reference sequences were aligned by ClustalW using the Mega 6 software [43] to identify potential multi-target primer binding sites. The designed primers (Table 1) were individually aligned to the representative sequence for each Diatrypaceous species (Table 2) and other fungal species associated with grapevine trunk diseases (Table 3) by ClustalW using the Mega 6 software [43], to manually check their theoretical specificity to their target species.

**Table 1:** Multi-target primers designed for quantitative PCR (qPCR) and High Resolution Melting Analysis (HRMA) used in this study.

Primer	Sequence	Target gene	Annealing temperature	Amplicon size (bp)	Method of analysis
DIA-17F DIA-122R	5’GGATCTCTTGGTTCTGGCAT3’ 5’ATGCCCACTAGAATACTAAT3’	5.8S	58°C	125	qPCR, Nested-PCR
DITS-1F DITS-1R	5’AAMCCATGTGAAYTTACCT3’ 5’CCAAGCARMTRGGGCTTGA3’	ITS1-ITS2	56°C	301–351	PCR, HRMA

**Table 2:** Fungal isolates used for the development of molecular tools to detect, quantify and identify Diatrypaceous species.

Species	Isolate ID	^a^Herbarium accession	Source [Reference]	DITS-1F and DITS-1R			DIA-17F and DIA-122R		
				PCR	^b^ Nested- PCR	^c^ HRMA	PCR	^d^ Nested-PCR	qPCR
*Cryptovalsa ampelina*	CSU01	n/a	[18]	+/-	**+**	**-**	**+**	**+**	**+**
	KC6	n/a	[18]	+/-	**+**	**-**	**+**	**+**	**+**
	PF5	n/a	^e^ SARDI	+/-	**+**	**-**	**+**	**+**	**+**
	B10-16A	n/a	[18]	+/-	**+**	**-**	**+**	**+**	**+**
	AD100	n/a	[18]	+/-	**+**	**-**	**+**	**+**	**+**
*C*. *rabenhorstii*	WA07CO	DAR81041	[18]	**+**		**+**	**+**	**+**	**+**
*Diatrypella vulgaris*	HVFRA04	DAR81031	[18]	**+**		**+**	**+**	**+**	**+**
	HVPT01	DAR81032	[18]	**+**		**+**	**+**	**+**	**+**
*Eutypa lata*	ADSC300	n/a	[18]	**+**	**+**	**+**	**+**	**+**	**+**
	ADSC400	n/a	[18]	**+**	**+**	**+**	**+**	**+**	**+**
	AHILLS	n/a	[18]	**+**		**+**	**+**	**+**	**+**
	B003	DAR79088	[44]	**+**			**+**	**+**	
	D03	n/a	SARDI	**+**			**+**	**+**	
	EP18	n/a	[18]	**+**			**+**	**+**	
	SACEA01	n/a	SARDI	**+**	**+**		**+**	**+**	**+**
	WB052	n/a	[44]	**+**	**+**	**+**	**+**	**+**	**+**
	OR039	n/a	[44]	**+**	**+**		**+**	**+**	**+**
*E*. *leptoplaca*	ADFIC100	n/a	[18]	**+**	**+**	**+**	**+**	**+**	**+**
	RGA02	n/a	[18]	**+**	**+**	**+**	**+**	**+**	**+**
	TUPN02	n/a	[18]	**+**	**+**		**+**	**+**	**+**
	TUQU01	n/a	[18]	**+**	**+**		**+**	**+**	**+**
	SAPN04	n/a	[18]	**+**			**+**	**+**	
	ABA200	n/a	[18]	**+**			**+**	**+**	
	ADSC500	n/a	[18]	**+**			**+**	**+**	
*Eutypella citricola*	T3R2S2	n/a	[18]	**+**	**+**	**+**	**+**	**+**	**+**
	WA06FH	n/a	[18]	**+**	**+**		**+**	**+**	**+**
	WA04LE	DAR81035	[18]	**+**	**+**	**+**	**+**	**+**	**+**
	WA05SV	DAR81036	[18]			**+**			
	TWD1	n/a	^f^ CSU	**+**			**+**	**+**	
	TWD2	n/a	CSU	**+**			**+**	**+**	
	TWD3	n/a	CSU	**+**			**+**	**+**	
*Eu*. *microtheca*	HVGRF02	DAR81039	[18]	**+**		**+**	**+**	**+**	**+**
	HVVIT05	DAR81040	[18]	**+**		**+**	**+**	**+**	**+**
*Eu*. *cryptovalsoidea*	HVFIG02	DAR81038	[18]	**+/-**		**-**	**+**	**+**	**+**

^a^ DAR—Agricultural Scientific Collections Unit, NSW DPI, Orange, Australia; (n/a)–not available

^b^ DNA were initially amplified using primers ITS1-ITS4 and the PCR products were subsequently used as template for the step 2 PCR using DITS-1F and DITS-1R

^c^ High Resolution Melting Analysis (HRMA)

^d^ DNA were initially amplified using primers DITS-1F and DITS-1R and the PCR products were subsequently used as template for the step 2 PCR using the primers DIA-17F and DIA-122R

^e^ SARDI–South Australian Research and Development Institute, Australia

^f^ CSU—Charles Sturt University, Wagga Wagga, NSW, Australia.

(+) positive amplification; (+/-) weak amplification; (-) negative amplification; (empty cells)–not tested

**Table 3:** Fungal isolates of non-target species used for testing the specificity of primers DITS-1F and DITS-1R and DIA-17F and DIA-122R using conventional PCR, quantitative PCR (qPCR) and High Resolution Melting Analysis (HRMA).

Species	Isolate	^a^ Herbarium accession	^b^ Disease	Source [Reference]	DITS-1F and DITS-1R		DIA-17F and DIA-122R	
					PCR	^e^ HRMA	PCR	qPCR
*Botryosphaeria dothidea*	BMV14	DAR79239	BD	[45]	-		-	-
	TS18	DAR79243	BD	[45]	-		-	
*Diplodia mutila*	CG15	DAR79135	BD	[45]	-	-	-	-
	CV5	DAR79129	BD	[45]	-		-	
	FF18	DAR79137	BD	[45]	-		-	
*D*. *seriata*	ME21	DAR79237	BD	[45]	-		-	
	A142a	DAR79990	BD	[46]	-	-	-	-
	C54a	DAR79996	BD	[46]	-		-	
*Dothiorella vidmadera*	M21	DAR78994	BD	[47]	-		-	
	L5	DAR78993	BD	[47]	-		-	
	J4	DAR78992	BD	[47]	-		-	
*Lasiodiplodia theobromae*	G31a	DAR77824	BD	[46]	-	-	-	-
	C2	DAR79507	BD	[45]	-		-	
	W200	DAR81024	BD	[48]	-		-	
*Neofusicoccum australe*	DNW8	DAR79501	BD	[45]	-	-	-	-
	FF10	DAR79502	BD	[45]	-		-	
	SDW4	DAR79505	BD	[45]	-		-	
*N*. *luteum*	H12-1	DAR80983	BD	[48]	-		-	
	HH119-1	DAR81013	BD	[48]	-	-	-	-
	BB175-2	DAR81016	BD	[48]	-		-	
*N*. *parvum*	B19A	DAR78998	BD	[45]	-	-	-	-
	B22a	DAR80004	BD	[46]	-		-	
	E12a	DAR77823	BD	[46]	-		-	
*Spencermartinsia viticola*	M11	DAR78868	BD	[45]	-		-	
	J8	DAR78867	BD	[45]	-		-	
	L19	DAR78870	BD	[45]	-		-	
	J7	DAR78869	BD	[45]	-		-	
*Phaeomoniella chlamydospora*	WB007	n/a	PD	^c^ SARDI	-		-	
	MA006	n/a	PD	SARDI	-		-	
	V21351a	V21351a	PD	G. Marchi	-		-	
	V22912	V22912	PD	I.G. Pascoe	-	-	-	-
*Phaeoacremonium minimum*	MA036	n/a	PD	SARDI	-		-	
	MA048	n/a	PD	SARDI	-		-	
	MA056	n/a	PD	SARDI	-	-	-	-
*Cadophora luteo-olivacea*	Clo2	n/a	PD	R. Baaijens	-	-	-	-
	Clo6	n/a	PD	R. Baaijens	-		-	
	Clo11	n/a	PD	R. Baaijens	-		-	
*Dactylonectria macrodidyma*	MW125	n/a	BF	M. Weckert	-		-	
	MW174	n/a	BF	M. Weckert	-	-	-	-
	MW580	n/a	BF	M. Weckert	-		-	
*llyonectria liriodendri*	MW65	n/a	BF	M. Weckert	-		-	
	MW122	n/a	BF	M. Weckert	-		-	
*Phomopsis viticola*	VRU006	n/a	PCL	C. Steel	-		-	
	VRU0076	n/a	PCL	C. Steel	-		-	
*Greeneria uvicola*	GR15	n/a	BR	[49]	-		-	
	GR35	n/a	BR	[49]	-		-	
*Colletotrichum acutatum*	CA15	n/a	BR	C. Steel	-		-	
	CA46	n/a	BR	C. Steel	-		-	
*Colletotrichum*. *gloeosporoides*	CG85	n/a	BR	C. Steel	-		-	
*Penicillium spp*.	PN1	n/a	BR	^d^ CSU	-		-	
*Botrytis cinerea*	TN118LS	n/a	BR	C. Steel	-		-	
	TN08065	n/a	BR	C. Steel	-		-	
*Alternaria sp*.	NT8	n/a	BR	CSU	-		-	
*Cladosporium sp*.	NT28	n/a	BR	CSU	-		-	
*Fusarium sp*.	MW887	n/a	Unspecified	M. Weckert	-		-	
	NT13	n/a	Unspecified	CSU	-		-	
*Conithyrium sp*.	NT6	n/a	Unspecified	CSU	-		-	
*Chaetomium sp*.	NT24	n/a	Unspecified	CSU	-		-	
*Epicoccum sp*.	EPI1	n/a	Unspecified	CSU	-		-	

^a^Agricultural Scientific Collections Unit, NSW DPI, Orange, Australia; (n/a)–not available

^b^ BD–Botryosphaeria dieback, PD–Petri Disease, BF–Black Foot, PCL–Phomopsis Cane and Leaf Spot, BR–Bunch Rot.

^c^ SARDI–South Australian Research and Development Institute, Australia

^d^ CSU—Charles Sturt University, Wagga Wagga, NSW, Australia.

^e^ HRMA–High resolution melting analysis (HRMA)

(-) negative amplification; (empty cells)–not tested

### DNA samples for the development of molecular tools

Total DNA of eight Diatrypaceae species (34 isolates) was obtained from the DNA collection at Charles Sturt University (CSU) and used in this study (Table 2). These isolates were previously identified by partial DNA sequencing of the rRNA and β-tubulin gene [18]. From the same CSU DNA collection, 59 fungal isolates of 26 non-target species isolated from grapevines including pathogens causing Botryosphaeria dieback (27 isolates), Petri disease (10 isolates) and Black foot (five isolates) were also used in this study (Table 3). All crude (non-purified) and genomic DNA were previously extracted from the pure cultures for each isolate using the PrepMan Ultra® (Applied Biosystems) or Qiagen DNeasy Plant DNA extraction kit (Qiagen, Australia), respectively, following the manufacturer’s protocols. The identity of these non-target isolates was confirmed by DNA sequencing of the ITS region of the ribosomal DNA [31].

### Primer optimisation, specificity and sensitivity tests by conventional PCR

The optimum annealing temperatures for the two multi-target primer pairs were determined using conventional PCR. The 25 μl PCR reactions contained 1× PCR buffer (Meridian Bioscience, USA), 1 μl of each primer (10 μm/μl), 1.25 U of MyTaq™ DNA Polymerase (Meridian Bioscience, USA) and 1 μl of gDNA of *C*. *ampelina* (CSU01), *E*. *lata* (ADSC300), and *Eu*. *citricola* (T3R2S2) as templates. Nuclease-free water was used as a non-template control (NTC). The PCR was set-up on a gradient block thermal cycler (C100™ Thermal Cycler, Bio-Rad Laboratories, Pty, Ltd.) using the following thermal cycling conditions: initial denaturation at 95°C for 5 min, followed by 35 cycles of 30 s at 94°C, 30 s annealing (gradient run of 54°C to 64°C) and 30 s at 72°C and a final extension of 72°C for 5 minutes. Following PCR amplification, 5 μl of each PCR product was visualised in a 1.0% agarose gel (Meridian Bioscience, USA) stained with 1× GelRed™ nucleic acid gel stain (Biotium, Hayward, CA, USA) using a Gel Doc XR+ Imaging System (Bio-Rad Laboratories, Pty, Ltd.). The optimum temperatures for each primer were further confirmed using qPCR.

The specificity of the primers was initially tested by conventional PCR using 1–2 ng of gDNA or crude DNA for all non-target species (Table 3). Conventional PCR was performed for each sample using the protocols described previously. Nuclease-free water was included as the NTC in each PCR.

The detection sensitivity for the different primers was further tested by conventional PCR. The gDNA of *C*. *ampelina* (CSU01), *E*. *lata* (ADSC300), and *Eu*. *citricola* (T3R2S2) was serially diluted from 10 ng/μl to 10 fg/μl and 1 μl of each dilution was used as a template using the optimum PCR reaction and thermal cycling condition previously described. The sensitivity of the primers was further assessed using nested-PCR. For DITS-1F and DITS-1R primers, the serially-diluted template DNA was initially amplified using the ITS1-ITS4 primers [50] following the same thermal cycling conditions previously described and 1 μl product from the first step PCR was used as a template for the second step PCR using multi-species primers DITS-1F and DITS-1R as previously described. For the DIA-17F and DIA-122R primers, the serially-diluted template DNA was initially amplified using DITS-1F and DITS-1R following the same thermal cycling conditions previously described and 2 μl product from the first step PCR was used as a template for the second step PCR using multi-species primers DIA-17F and DIA-122R as previously described.

### Quantitative PCR using SYBR green

To test the primers DIA-17F and DIA-122R, all qPCRs were carried out in 0.1 ml strip tubes and caps (Qiagen, USA) using a RotorGene 6000 (Corbett Life Science, Qiagen, USA). The 20 μl qPCR reaction contained 10 μl of 2× SsoAdv Universal SYBR Green Supermix (Bio-Rad, USA); 1 μl each of forward and reverse primer (10 μm/μl) and 2 μl gDNA of *C*. *ampelina* (CSU01), *E*. *lata* (ADSC300), and *Eu*. *citricola* (T3R2S2) were used as templates. The qPCR conditions were set-up using the optimal annealing temperature and primer concentration as determined previously. All qPCR reactions were conducted using four replicates in three independent assays using the following thermal cycling conditions: 3 mins at 98°C, followed by 35 cycles of 98°C for 30 s, 58°C for 15 s and fluorescence detection at 58°C for 15 s. After the final amplification cycle, a dissociation (melting) curve (72°C to 95°C) was generated to check the specificity of the amplification reaction. For each assay, three controls were included: a) NTC (nuclease-free water); b) positive control (*E*. *lata* gDNA, 2 ng); and c) negative control (*Neofusicoccum luteum* gDNA, 2 ng).

The results of the specificity test for DIA-17F and DIA-122R primers using conventional PCR was further confirmed by qPCR using ~2 ng of gDNA of representative non-target species (Table 3). All qPCRs were performed in four replicates using the protocols described previously. For each qPCR assay, three controls were included: (1) 2 pg Diat-5S gBlock as standard; (2) NTC (nuclease-free water); and (3) *E*. *lata* gDNA (2 ng) for the positive control.

The efficiency of the qPCR using DIA-17F and DIA-122R primers was further determined using 10-fold serial dilutions of two different templates: (a) gDNA of Diatrypaceous species, *C*. *ampelina* (CSU01), *E*. *lata* (ADSC300), *E*. *leptoplaca* (RGA02) and *Eu*. *citricola* (T3R2S2); and (b) chemically synthesized DNA fragments (gBlocks^®^ Gene Fragments Integrated DNA Technologies, USA). The 460 bp (Diat-5S) gBlocks^®^ (Integrated DNA Technologies, USA, S1 Fig) was designed based on the *E*. *lata* ITS sequences and shared sequence homology with DIA-17F and DIA-122R primers (S1 Fig). The 500 ng gBlocks^®^ were resuspended in 50 μl of TE Buffer (Tris and EDTA, pH 8.0; Sigma Aldrich) following the manufacturer’s recommendation, to reach a final concentration of 10 ng/μl and used as qPCR standards. The mass of the Diat-5S gBlock was calculated as 0.50 attograms (ag)/copy (0.50e-18/copy) which is equivalent to 2.2 × 10^9^ copies/ng of Diat-5S gBlocks.

The quantification cycle (Cq) value for each standard sample was calculated and analysed using the Rotor-Gene Q Series software (Version 2.3.1) to generate a standard curve. The number of copies for each Diat-5S gBlock standard dilution was plotted against the Cq values and the resulting linear regression equations were used to quantify the number of copies of each target gene in the unknown samples. The limit of detection (LOD) for each standard was determined following the MIQE guidelines [51] which was based on the number of copies required to obtain reproducible detection in three consecutive assays.

The number of rRNA gene copies in a Diatrypaceae genome is unknown making it difficult to calculate spore numbers based on the number of rRNA copies amplified by qPCR. Since the size of the *E*. *lata* haploid genome (or one spore) is 54 mbp or ~54 fg [52], the average rDNA copies per ~54 fg DNA for the four representative species was determined using 10-fold serial dilutions of *C*. *ampelina* (CSU01), *E*. *lata* (ADSC300), *E*. *leptoplaca* (RGA02) and *Eu*. *citricola* (T3R2S2) gDNA. Each gDNA dilution (2 ng to 20 fg) was analysed by qPCR with Diat-5S gBlock standards using four replicates in three independent assays following the conditions previously described. The quantification of target rRNA gene per nanogram of DNA was achieved by comparing the Cq values for each gDNA concentrations with the Cq values of the Diat-5S gBlock standards. The average rDNA copies per ~54 fg DNA for each representative four species were determined and subsequently used for calculating the number of spores detected in spore trap DNA samples.

### High-resolution melting analysis (HRMA)

The PCR primers DITS-1F and DITS-1R that can amplify bigger fragments (305–350 bp) of the ITS region of different Diatrypaceous species were further tested for their suitability for HRMA. The different sizes and GC content of each target Diatrypaceous species will potentially generate unique melting profiles that will allow identification of individual target species using the multi-target primers.

To evaluate the suitability of HRMA to identify Diatrypaceous species, 1 ng/μl gDNA from pure cultures of *C*. *ampelina* (CSU01), *C*. *rabenhorstii* (DAR81041), *D*. *vulgaris* (DAR 81031), *E*. *lata* (ADSC300), *E*. *leptoplaca* (RGA02) *Eu*. *citricola* (T3R2S2), and *Eu*. *microtheca* (DAR81039), was prepared and 2 μl of each sample was used for PCR amplification. The gDNA (1–2 ng) of representative non-target species (Table 3) were included in the preliminary tests to further confirm the specificity of the primers using HRMA. The PCR amplification, DNA melting and fluorescence data acquisition were performed in 0.1 ml strip tubes and caps (Qiagen, USA) using a RotorGene 6000 (Corbett Life Science, Qiagen). The 25 μl reaction mix contained 12.5 μl of 2× Type-it® HRM PCR master mix (Qiagen, USA); 1.75 μl (10 μm/μl) of each primer, 2 μl template and nuclease-free water. The PCR was carried out using the following the manufacturer’s recommended protocol for microbial genetic difference [53]: initial PCR activation step at 95°C for 5 min, followed by 40 cycles of 95°C for 10 s, 55°C for 30 s and fluorescence data acquisition at 72°C for 10 s. After the final amplification cycle, HRMA was carried out using Rotorgene Q series software ver.2.3.4 at a temperature ramping from 65°C to 95°C set to increase at 0.1°C per 2 s each step. The melting curves were normalised by selecting the baseline data for the pre-melt and post-melt phases. Melt profiles for each sample were visualised and compared using the normalised and difference graphs. In the difference graph, the melt profiles for each species were compared against each other by converting the profile of one species to a horizontal line. The assay was repeated using gDNA of two to three isolates of each Diatrypaceous species to determine the reproducibility of the method (Table 2).

To determine the effects of DNA concentrations on melting profiles, HRMA was also carried out using different concentrations of gDNA (1 ng, 200 pg, 100 pg, 50 pg) of *D*. *vulgaris* (DAR 81031), *E*. *lata* (ADSC300), *E*. *leptoplaca* (RGA02) *Eu*. *citricola* (T3R2S2), and *Eu*. *microtheca* (DAR81039) using the methods described previously. The 1 ng template was used as the reference sample for each species for the HRMA. Due to the low expected concentrations of fungal DNA from each spore tape DNA sample, the lowest DNA concentration that generated a melt profile for each reference species that can be distinguished at 90% confidence was used as the concentration for standards in the subsequent assays.

### Surveillance of Diatrypaceous airborne spores

#### DNA samples

The DNA from airborne spore samples used in this study was obtained from two spore surveillance studies conducted in different wine growing regions in Australia. Volumetric spore traps (Burkard Manufacturing Co, Rickmansworth, England) fitted with a battery and solar panel for recharging (Measurement Engineering Australia) were used for spore trapping [31].

For the first spore surveillance, one spore trap was deployed in each of the Barossa Valley and Coonawarra, South Australia (SA) and the Hunter Valley and Riverina, New South Wales (NSW) from 2014–2016 (S2 Fig). For the second surveillance conducted in 2017–2021, only the Barossa Valley and Coonawarra spore traps were continued while one trap each were deployed in the Adelaide Hills, Clare Valley and McLaren Vale, South Australian wine regions, and in Tumbarumba, NSW (S2 Fig). The cultivars, collection dates, climatic conditions for each region are summarised in S1 Table.

All spore trap deployment, spore tap sample collection and DNA extractions for the two surveillance studies were carried out following the methods developed for Botryosphaeriaceae spore surveillance studies [31]. The tapes were removed from the drum, cut longitudinally into two pieces. Total DNA was extracted from each tape using the modified Gentra Puregene protocol [31]. To check for any variations in each batch of DNA extractions, two control tapes were prepared: (1) non-inoculated tape (non-template control) and (2) tape inoculated with 1000 *E*. *lata* ascospores (positive control). Each control tape was placed inside a 2 ml lysing tube [31] and stored in -20°C until processing. One tube each of the control tapes was included in each batch of DNA extraction.

#### Detection and quantification of Diatrypaceous inoculum

BLAST analysis of the forward primer DIA-17F revealed high sequence similarities with other fungal species. Although specificity tests showed other trunk disease pathogens were not amplified using these primers, all spore tape DNA samples were initially tested for the presence or absence of Diatrypaceous DNA by nested-PCR using two sets of Diatrypaceous multi-target primers to reduce the risk of any off-target amplification. The nested-PCR was carried out by initially amplifying spore tape DNA extracts (2 μl) but using 0.5 μl for each of the DITS-1F and DITS-1R primers (Step 1) followed by DIA-17F and DIA-122R (Step 2) using 1 μl product from the first step PCR. For each nested-PCR, four controls were included: a) NTC (H_2_O); b) non-inoculated spore tape; c) *N*. *luteum* conidia inoculated tape (negative control); and d) *E*. *lata* ascospore inoculated tape (positive control). PCR products from the second step of each nested PCR were visualised by electrophoresis following the protocol described previously.

All samples that tested positive to nested PCR were further analysed by qPCR using the primers DIA-17F and DIA-122R to determine the number of spores trapped in each 2-day period for all spore tape samples. Each qPCR was performed following the protocols described previously and the following controls and standards were included: a) Diat-5S gBlock standard solutions (2 pg); b) NTC (nuclease-free water); c) non-inoculated tape control; and d) *N*. *luteum* inoculated tape; e) *E*. *lata* inoculated tapes. All samples were analysed in duplicates except for the NTC and gBlock standards that were analysed in four replicates.

To analyse samples for each qPCR run, the standard curves previously developed were imported into the Rotor-Gene Q Series software (Version 2.3.1). The standards Diat-5S gBlocks that were included in each run were used to calibrate the imported standard curve. The mean Cq values for each unknown sample were plotted against the Cq values of the standards and the resulting regression equations were used to quantify the number of spores in each sample as previously described.

### Data analyses

To calculate the number of Diatrypaceous spores in 2-day spore tape samples, a published formula [32] with some modifications was used:

N=(Q/110)(D/T)(2).

Where:

*N* = Calculated no. of spores in a 2-day period,

*Q* = the mean copies of rDNA from 2-day period detected by qPCR,

110 = calculated no. of rDNA copies in a single Diatrypaceous ascospore. This value was calculated based on the size of the *E*. *lata* haploid genome (or one spore) which is 54 mbp or ~54 fg [52] and the average rDNA copies amplified by qPCR per ~54 fg Diatrypaceous DNA.

*D* = the total DNA volume (30 μl) extracted from a ½ section of a 2-day sample,

*T* = the amount of DNA template (2 μl/reaction) used in one qPCR reaction,

2 = the total number of tape sections for the 2-day collection period.

For spore surveillance, the average number of Diatrypaceous airborne spores detected in all 2-day samples for each month from each region were calculated. The frequency of spore detection was plotted with the total rainfall for the same period for each location to determine the correlation between spore release and rainfall. The weather data for each spore surveillance site were sourced from the Bureau of Meteorology (www.bom.gov.au) except for Tumbarumba, NSW that was sourced from the NSW Department of Primary Industry weather station (https://www.dpi.nsw.gov.au/agriculture/horticulture/ grapes/weather-stations-network/wsn).

### High resolution melting analysis (HRMA) and DNA sequencing of spore tape DNA samples

HRMA was further carried out to identify the airborne Diatrypaceous spores trapped in different vineyards. Spore tape DNA samples (20–25 samples each) that tested positive to qPCR were randomly selected from different sample dates from each region. The DNA samples were pre-amplified by conventional PCR prior to HRMA to increase sensitivity and the amount of template required for the assays. Each selected sample was pre-amplified using the primers ITS1 and ITS4 [50] using the PCR reactions and thermal cycling conditions described previously, and the subsequent PCR products were diluted to 1:4 and 2 μl of each diluted sample was used as templates for HRMA. For each assay, 200 pg gDNA (minimum DNA concentration that can generate a clear and strong melt profile) extracted from pure cultures for *E*. *lata* (ADSC300), *E*. *leptoplaca* (RGA02), *Eu citricola* (T3R2S2) and *Eu*. *microtheca* (DAR 81039) was included as reference samples. All samples were analysed in duplicates and nuclease-free water was used as non-template controls.

The HRMA was performed using the Rotor-Gene Q Series software (Version 2.3.1). The reaction efficiency for each sample was determined using the Comparison quantification tool and any sample with an efficiency of less than 1.4 was considered an outlier and was excluded in the analysis as recommended by the manufacturer [54]. The melting profile for each unknown sample was visualised using normalised and difference graphs using the known Diatrypaceous species as reference genotypes. Each unknown sample was plotted with the reference Diatrypaceous species and assigned to a genotype at a confidence value of ≥70% while samples with the confidence value of <70% were assigned as variants. For samples that fell below the 70% confidence threshold, each melt profile was visually-inspected and any ambiguous profiles were retested to confirm the results.

To verify the results of the HRMA, the same spore tape samples were further analysed by DNA sequencing. To increase the amount of DNA required for DNA sequencing, all selected samples that were initially amplified using the primers ITS1-ITS4 [50] as described in the HRMA were used as a template for the second PCR using multi-species primers DITS-1F and DITS-1R as previously described. All selected PCR products were purified using the Favorgen PCR purification kit (Favorgen Biotech Corp., Taiwan) following the manufacturer’s protocols. All purified products were quantified using fluorometry (Quantus, Promega, USA) and 10–12 ng of purified DNA were sent to the Australian Genome Research Facilities (AGRF, Sydney, NSW) for one-way Sanger sequencing using the DITS-1F primer. The resulting sequences and chromatographs were analysed using DNAMAN 5.2 (Lynnon Biosoft^©^) and Chromas Lite 2.1^©^ (Technelysium PTY Ltd) software and edited when necessary. All trimmed DNA sequences were subsequently identified using the Basic Local Alignment Search Tool (BLAST) in the GenBank (https://www.ncbi.nlm.nih.gov/genbank). The species was considered a match if it had 100% homology with a continuous section of at least 270 bp of the ITS region of the reference species.

Following the results of the BLAST, a phylogenetic analysis was further carried out for the sequences of two new species not previously reported in Australian vineyards. Published sequences of the ribosomal DNA for *Cryptosphaeria* sp. (Accession no. HQ692618.1), *Diatrype stigma* (Accession no. KU170618.1), *D*. *brunneospora* (Accession No. HM581946.1*)* and *Diatrype* spp. (Accession No. HQ692618.1), reported in Australia [18], *Cryptosphaeria multicontenintalis* (Accession no. MF359629.1) and *E*. *lata* (Accession no. HQ692611.1) were obtained from the NCBI database (www.ncbi.nlm.nih.gov) and aligned with the sequences obtained from spore tape samples using MEGA 6 software [43]. One rDNA sequence for *C*. *ampelina* (Accession no. KY849963.1) was further obtained from the NCBI database and included as an outgroup. The aligned sequences were tested for phylogeny and a neighbour joining tree was generated. The robustness of the trees obtained was evaluated by 1000 bootstrap replicates [55].

## Results

### Primer development and optimisation

Alignment of the rDNA sequences of representative Diatrypaceous species reported in Australia identified two binding sites for multi-species primers suitable for qPCR (Table 1). The Diatrypaceae multi-species primer DIA-17F and DIA-122R were designed to anneal to the 5.8S rRNA gene region for all eight target Diatrypaceous species. Preliminary tests using conventional PCR showed the optimum annealing temperature for these primers was 58°C which amplified a single PCR product of 125 bp for all eight Diatrypaceous species tested (Table 2). No PCR products were observed with the DNA from nine Botryosphaeriaceae species (27 isolates) and 17 other non-target species (32 isolates) from grapevines (Table 3).

Alignment of the rDNA sequences further identified two binding sites for multi-species primer suitable for conventional PCR and HRMA (Table 1). The Diatrypaceae multi-species forward primer DITS-1F is degenerate at nucleotides 3 and 13 and is designed to anneal to the ITS-1 region, while the reverse primer DITS-1R is degenerate at nucleotides 8, 9 and 11 and is designed to anneal to the ITS-2 region of *C*. *rabenhorstii*, *Diatrypella vulgaris*, *E*. *lata*, *E*. *leptoplaca*, *Eu*. *citricola* and *Eu*. *microtheca*. However, the reverse primer DITS-1R has two base mismatch with *C*. *ampelina* and *Eu*. *cryptovalsoidea*. Preliminary tests using conventional PCR showed the optimum annealing temperature for the multi-species primers DITS-1F and DITS-1R was 56°C which amplified a single PCR product of different sizes (S3 Fig) for all six target species: *C*. *rabenhorstii* (301 bp), *D*. *vulgaris* (351 bp), *E*. *lata* (350 bp), *E*. *leptoplaca* (351 bp), *Eu*. *citricola* (327 bp) and *Eu*. *microtheca* (313 bp) while *C*. *ampelina* (350 bp) was weakly amplified using ~20 ng of gDNA (Table 2). The nested-PCR using DITS-1F and DITS-IR primers (Step 1) and DIA-17F and DIA-122R (Step 2) was able to detect ~10 fg of *E*. *lata*, *E*. *leptoplaca*, and *Eu*. *citricola* gDNA while the nested-PCR was able to detect only ~500 pg of *C*. *ampelina* DNA. No PCR products were observed with the nine Botryosphaeriaceae species (27 isolates) or 17 other non-target fungal species (32 isolates) from grapevines, indicating that the primers were specific to the target Diatrypaceae species tested (Table 3).

### Quantitative PCR for the Diatrypaceous spores

The qPCR with the primer pair DIA-17F and DIA-122R (Table 1) using SYBR green successfully amplified Diat-5S gBlock and the gDNA of eight Diatrypaceous pathogens previously reported in Australian vineyards (Table 2). A reaction efficiency of 99% and 95% with coefficients of determination of *R*^2^ *=* 0.997 and *R*^2^ *=* 0.980–0.997 (Tables 4 and 5) were obtained based on the slopes of standard curves of the Diat-5S gBlock and gDNA, respectively. The analysis of the melt curve showed that the DIA-17F and DIA-122R primers produced unique amplicons for both the Diat-5S gBlocks and gDNA that resulted in a single peak at 83.5°C (±0.3) while no peaks were observed with NTC (H_2_O) and non-target DNA (*N*. *luteum*). The qPCR using serially diluted Diat-5S gBlocks and gDNA of *C*. *ampelina* (CSU01), *E*. *lata* (ADSC300), *E*. *leptoplaca* (RGA02), and *Eu*. *citricola* (T3R2S2) demonstrated that the qPCR primers could detect as low as 20 copies of gBlocks (Table 4) and 20 fg gDNA (Table 5), respectively. No fluorescence detection was observed with any of the representative DNA of the Botryosphaeriaceae species or other non-target fungal species known to be present in Australian vineyards (Table 3).

**Table 4:** Determination of the limit of detection (LOD) of the qPCR using different concentrations of Diat-5S gBlocks®.

Copies/reaction	^a^ Cq values	^b^ Signal ratio
**4,000,000**	10.63 ± 0.08	12/12
**400,000**	14.29 ± 0.07	12/12
**40,000**	18.39 ± 0.40	12/12
**4,000**	21.51 ± 0.25	12/12
**400**	24.39 ± 0.18	12/12
**40**	27.46 ± 0.65	12/12
**20**	29.28 ± 0.78	12/12
**10**	No detection	0/12

^a^ Quantification cycles (Cq) at which fluorescence was detected. The Cq values and corresponding standard errors (±) were derived from the means of three independent assays each with four technical replicates. The coefficient of determination (*R*^*2*^) was 0.997.

^b^ Number of positive samples out of the total number of reactions.

**Table 5:** Determination of the limit of detection (LOD) of the qPCR using different concentrations of genomic DNA of *Eutypa lata*, *Cryptovalsa ampelina*, *Eutypella citricola* and *E*. *leptoplaca*.

fg/reaction	^a^ Cq values ± Std Dev	^b^ Signal ratio
***C*. *ampelina* (CSU01)**		
** 5,000,000**	10.09 ± 0.00	8/8
** 500,000**	13.41 ± 0.02	8/8
** 50,000**	16.82 ± 0.12	8/8
** 5,000**	20.16 ± 0.01	8/8
** 500**	23.82 ± 0.32	8/8
** 50**	27.37 ± 0.82	8/8
***Eutypa lata* (ADSC300)**		
** 2,000,000**	13.79 ± 0.22	8/8
** 200,000**	16.15 ± 0.51	8/8
** 20,000**	19.60 ± 0.22	8/8
** 2,000**	24.54 ± 0.14	8/8
** 200**	26.34 ± 0.46	8/8
** 20**	30.42 ± 1.57	8/8
***E*. *leptoplaca* (RGA02)**		
** 2,200,000**	11.47 ± 0.03	8/8
** 220,000**	14.97 ± 0.03	8/8
** 22,000**	18.36 ± 0.07	8/8
** 2,200**	24.07 ± 0.14	8/8
** 220**	24.35 ± 062	8/8
** 22**	29.50 ± 1.31	4/4
***Eutypella citricola* (T3R2S2)**		
** 4,500,000**	10.42 ± 0.02	8/8
** 450,000**	13.92 ± 0.09	8/8
** 45,000**	17.33 ± 0.04	8/8
** 4,500**	21.97 ± 0.03	8/8
** 450**	24.80 ± 0.20	8/8
** 45**	28.92 ± 0.21	8/8

^a^ Quantification cycles (Cq) at which fluorescence was detected. The Cq values and corresponding standard deviations (±) were derived from the means of two independent assays each with four technical replicates. The coefficients of determination (*R*^*2*^) were *C*. *ampelina*—0.997; (1) *E*. *lata*—0.983; (b) (c) *E*. *leptoplaca*– 0.980 and (d) *Eu*. *citricola*—0.997.

^b^ Number of positive samples out of the total number of reactions.

The calculated number of copies of rRNA gene per ng of gDNA of the four representative Diatrypaceous pathogens by qPCR are presented in Table 6. Using these estimates, the average number of copies of the rRNA gene in a haploid genome (54 mbp or ~54 fg) [52] for the four representative Diatrypaceae species ranged from 105–114 and the mean value of 110 was used to estimate the number of spores in spore tape samples from vineyards. Since the LODs for the qPCR was 20 copies and 20 fg, using the Diat-5S gBlock standard and gDNA, respectively, this is equivalent to less than one spore.

**Table 6:** The calculated number of copies of the ribosomal RNA (rRNA) gene in one haploid genome of the four representative Diatrypaceae species.

Species	Mean no. of rRNA gene copies /ng of gDNA	^a^ No. of haploid genome (~54 fg) per ng gDNA	No. of rRNA gene copies in a haploid genome
***Cryptovalsa ampelina* (CSU01)**	1,942,500	18,518.5	105
***Eutypa lata* (ADSC300)**	2,039,750	18,518.5	110
***E*. *leptoplaca* (RGA02)**	2,119,115	18,518.5	114
***Eutypella citricola* (T3R2S2)**	2,046,464	18,518.5	111
**Mean**	2,036,957	18,518.5	110

^a^The calculated number of rRNA gene copies was based on the means of four technical replicates in two independent assays that were plotted against the Diat-5S gBlock gene fragments used for standard curves. The resulting linear regression equations were used to quantify the number of copies of the rRNA gene per ng of gDNA for each species tested. The number of rRNA gene copies in one spore was calculated based on the estimated size of *Eutypa lata* haploid genome of 54 mbp (~54 fg) [56].

#### High resolution melting analysis (HRMA)

**Fig 2:**
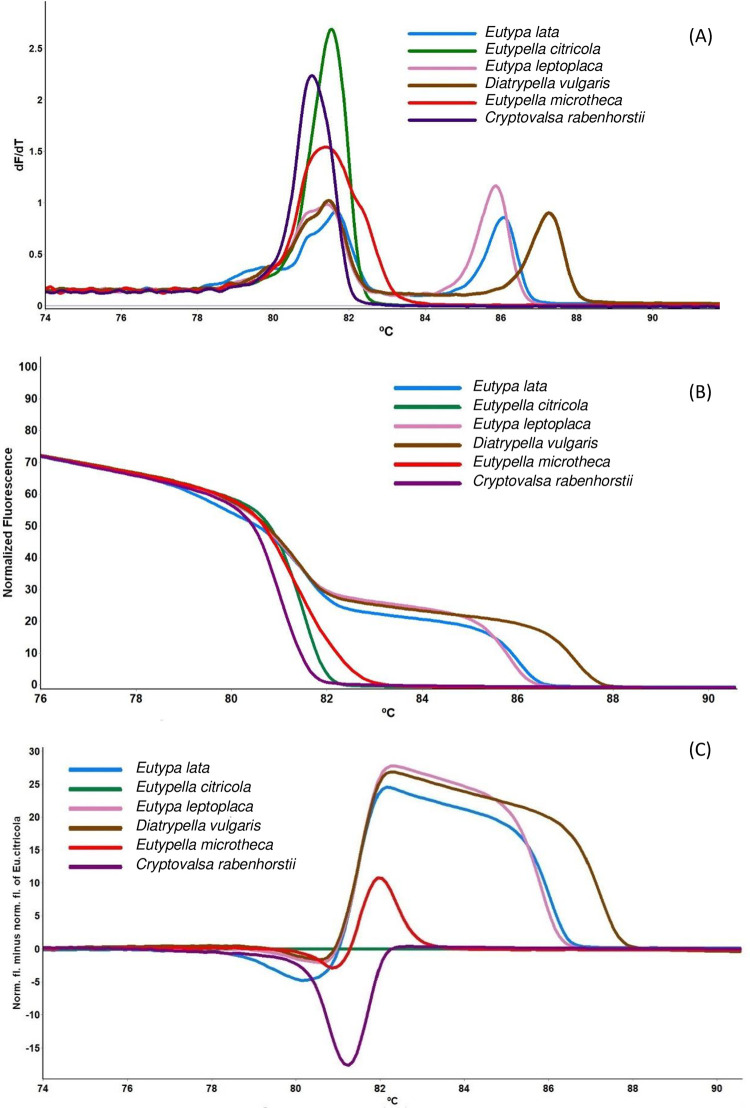
High resolution melting analysis (HRMA) for the identification of Diatrypaceous species in Australian vineyards. (A) melt curve profiles; (B) normalised graph; (C) difference graph with *Eutypella citricola* as the core genotype which was converted to a horizontal line.

All representative isolates for *C*. *rabenhorstii*, *D*. *vulgaris*, *E*. *lata*, *E*. *leptoplaca*, *Eu*. *citricola*, and *Eu*. *microtheca*, were successfully amplified using the primers DITS-1F and DITS-1R, thus, normalised and difference melt profiles were produced (Fig 2). However, *C*. *ampelina* and *Eu*. *cryptovalsoidea* were not successfully amplified due to high sequence dissimilarities with the primer DITS-1R. No fluorescence detection was further observed with any of the representative DNA of the Botryosphaeriaceae species or the other non-target fungal species known to be present in Australian vineyards (Table 3).

The visual inspections of the melting curve for each representative Diatrypaceous species revealed six unique and distinct melt profiles. The melt profiles for *D*. *vulgaris*, *E*. *lata* and *E*. *leptoplaca* with bigger PCR products (305–351 bp) yielded two distinct peaks, while *C*. *rabenhorstii*, *Eu*. *citricola*, *Eu*. *microtheca* with small PCR fragments (301–327 bp) yielded one simple single-peak melting profile (Fig 2A). The gel electrophoresis from the PCR products using DITS-IF and DITS-1R confirmed the presence of a single band (S3 Fig) for all Diatrypaceous species tested indicating that the complex HRMA melt curves observed for *D*. *vulgaris*, *E*. *lata* and *E*. *leptoplaca* were derived from a single amplicon and not due to non-specific amplification.

The normalised HRMA curves of six representative Diatrypaceous species (Fig 2B) further revealed all six species could be visually distinguished based on the shape of their melt profile. When the melt profiles for each species were compared, the difference graph which converted *Eu*. *citricola* as a horizontal line (Fig 2C) revealed clear distinct profiles that can discriminate the six species at a confidence value of 99%, making this species a suitable core genotype for identifying unknown samples using HRMA. The minimum DNA concentration that generated a distinguishable melt profile at >90% confidence was 200 pg while the shapes of the curves flattened at lower DNA concentrations due to low fluorescence signal.

### Diatrypaceous airborne inoculum

#### 2014–2016 monitoring

**Fig 3:**
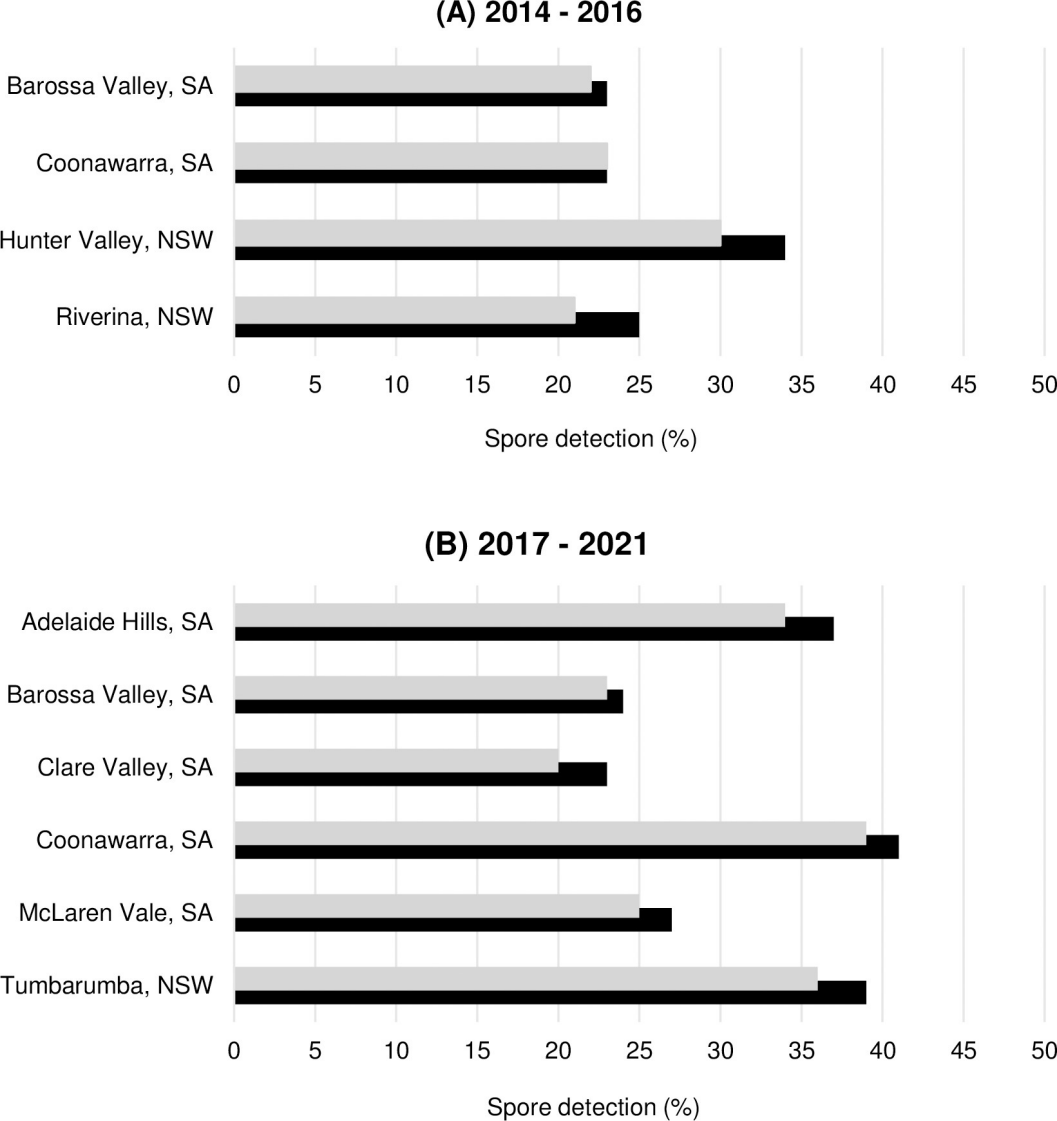
Detection of Diatrypaceous spores from 2-day samples from different wine growing regions in South Australia (SA) and New South Wales (NSW) during the 2014–2016 (A) and 2017–2021 (B) spore surveillance studies. Black bars (■) are the total samples that tested positive to Diatrypaceous spores and grey bars (■) are the proportion of the positive samples that were trapped during or within 48 hours of a rain event.

A total of 1,796 spore tape samples (2-day periods) collected between 2014–2016 for each region were analysed using molecular tools, and 473 samples (26%) tested positive to Diatrypaceous spores (S2 Table). The highest spore detection was recorded in the Hunter Valley, NSW in 34% of the assessed samples, while the spore detection for the other three regions was 23–25% (Fig 3A, S2 Table). At all four sites, Diatrypaceous spores were generally trapped during or immediately after rainfall particularly in Coonawarra, SA where all spore detection coincided with rain events (Fig 3A). However, on a few occasions, Diatrypaceous spores were also detected in the absence of rain, (i.e. at least 4 days before or after a rain event), particularly in the Riverina and the Hunter Valley, NSW (Fig 3A).

**Fig 4:**
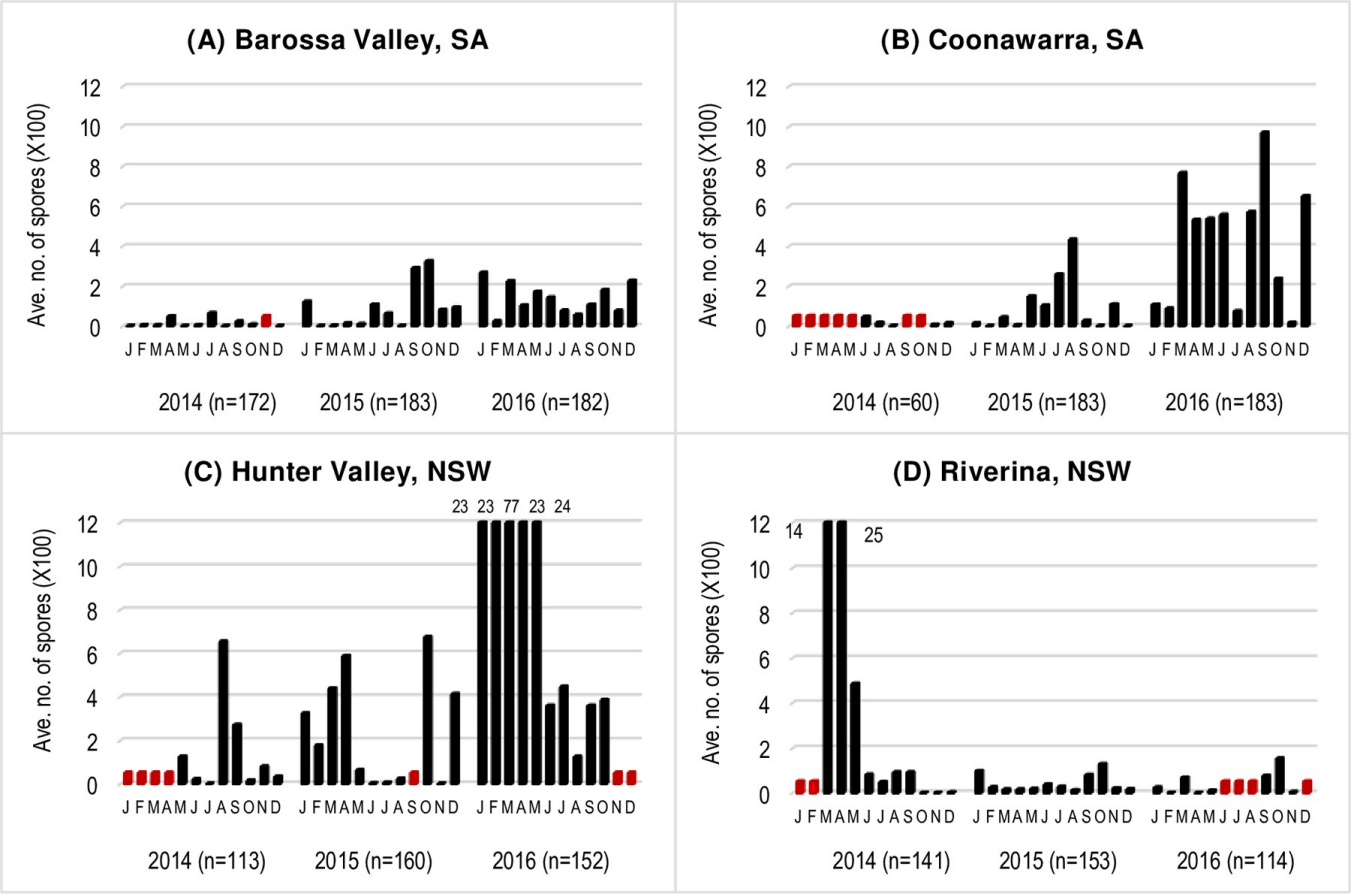
Average number of Diatrypaceous spores detected in all 2-day samples for each month in different wine growing regions in Australia from 2014–2016. Differences in the number of samples (n) between years and regions were due to the missing data (red bars) associated with spore trap deployment dates or spore trap malfunctions.

For the 2014–2016 spore monitoring, the average number of Diatrypaceous airborne spores detected in 2-day samples for each month from each region are summarised in Fig 4. Overall, Diatrypaceous spores were detected in all regions, but spore numbers and detection frequency varied between regions, years and seasons.

From the Barossa Valley, SA, analyses of the 537 spore tape samples collected from January 2014 to December 2016, revealed 126 samples (23%) tested positive to Diatrypaceous spores (Fig 3A; S2 Table). The highest spore detection was recorded in 2016 (n = 67) and the lowest in 2014 (n = 8) (Fig 4A). The average number of spores detected in all 2-day samples for each month ranged from 4 to 324 with the highest number detected in October 2015 (Fig 4A). The spore trap malfunctioned for 22 days in 2014 and reduced the number of samples collected during this period.

From the Coonawarra, SA, analyses of the 426 spore tape samples collected from January 2014 to December 2016, revealed 100 samples (23%) tested positive to Diatrypaceous spores (Fig 3A, S2 Table). The highest spore detection was recorded in 2016 (n = 66) and lowest in 2014 (n = 6). The average number of spores detected in all 2-day samples for each month ranged from 7 to 968 with the highest number of spores detected in September 2016 (Fig 4B). The number of samples for 2014 was low since the spore trap malfunctioned for 7 months and reduced the number of samples collected during this period.

From the Hunter Valley, analyses of the 425 samples collected from May 2014 to November 2016, revealed 145 samples (34%) tested positive to Diatrypaceous spores (Fig 3A; S2 Table). The highest spore detection was recorded in 2016 (n = 83) and lowest in 2014 (n = 22). The average number of spores detected in all 2-day samples each month ranged from 15 to 7668. In 2016, the average number of spores detected in all 2-day samples for each month was extremely high in January to May with an average of 2251 to 7668 spores during this 5-month period (Fig 4C). The number of samples for 2014 was lower since the spore trapping commenced in May 2014.

From the Riverina, NSW, analyses of the 408 spore tape samples collected from June 2014 to November 2016, revealed 102 samples (25%) tested positive to Diatrypaceous spores (Fig 3A; S2 Table). The highest spore detection was recorded in 2016 (n = 37) and lowest to 2014 (n = 30). The average number of spores detected in 2-day samples for each month ranged from 3 to 2534 with highest average number of spores detected in all 2-day samples in March (1,358 spores) and April (2,534 spores) 2014 (Fig 4D). The spore trap was installed in March 2014 reducing the samples during that period. The spore trap also malfunctioned for four months in 2016 resulting in a lower number of samples collected during that year.

### 2017–2021 spore monitoring

A total of 5089 spore tape samples collected in 2017–2021 for all sites were analysed and 1609 (32%) of the 2-day samples tested positive to Diatrypaceous spores (S3 Table). The highest spore detection was recorded in the Coonawarra at 41% and lowest in Clare Valley, SA at 23% (Fig 3B; S3 Table). Similar to the results from the first spore surveillance (Fig 3B), Diatrypaceous spores were generally trapped during or immediately after the occurrence of rain (Fig 3B). However, on few occasions Diatrypaceous spores were also trapped in the absence of rain (i.e. at least 4 days before or after a rain event for all six sites (Fig 3B).

**Fig 5:**
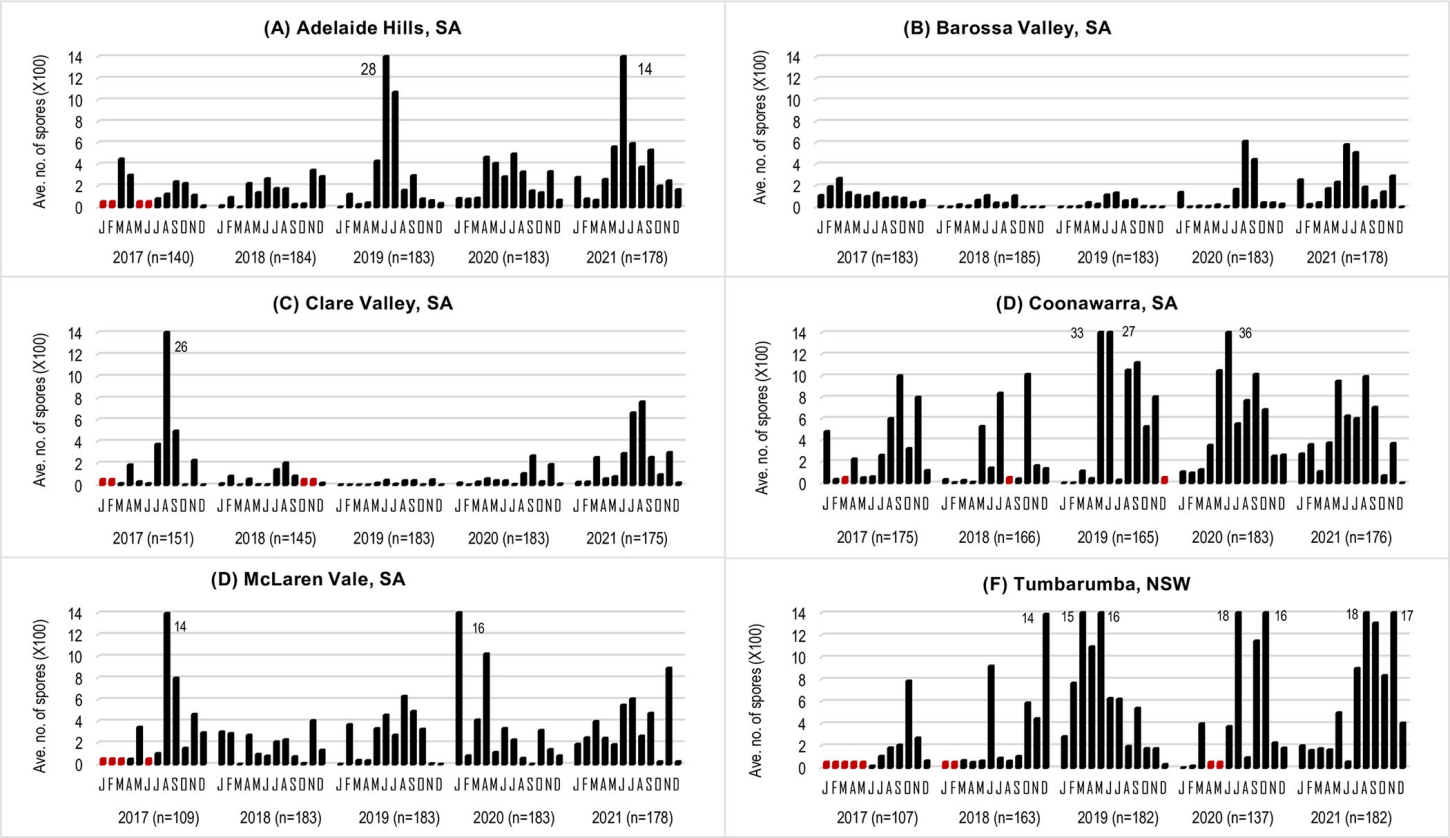
Average number of Diatrypaceous spores detected in all 2-day samples for each month in different wine growing regions in Australia from 2017–2021. Differences in the number of samples (n) between years and regions were due to the missing data (red bars) associated with spore trap delopyment dates or spore trap malfunctions.

For the 2017–2021 spore monitoring, the average number of Diatrypaceous airborne spores detected in all 2-day samples for each month from each region are summarised in Fig 5. Similar to the first surveillance, Diatrypaceous airborne spores were detected sporadically at different times of the year with the spore detection and numbers varying between regions and years.

From the Adelaide Hills, analyses of the 868 spore tape samples collected from March 2017 to December 2021, revealed 322 samples (37%) tested positive to Diatrypaceous spores (Fig 3B; S3 Table). The highest spore detection was recorded in 2021 (n = 105) and lowest in 2017 (n = 38). The average number of spores detected in all 2-day samples for each month ranged from 10 to 2,800 with the highest number detected in June 2019 (Fig 5A).

From the Barossa Valley, analyses of the 912 spore tape samples collected from January 2017 to December 2021, revealed 222 (24%) of the samples tested positive to Diatrypaceous spores (Fig 3B; S3 Table). The highest spore detection was recorded in 2021 (n = 69) and the lowest in 2018 (n = 28). The average number of spores detected in all 2-day samples for each month ranged from 1 to 609 with the highest number in August 2020 (Fig 5B).

From the Clare Valley, analyses of the 837 spore tape samples collected from March 2017 to December 2021, revealed 192 (23%) of the samples tested positive to Diatrypaceous spores (Fig 3B; S3 Table). The highest spore detection was recorded in 2021 (n = 104) and the lowest in 2018 (n = 14) and 2019 (n = 17). The average number of spores detected in all 2-day samples for each month ranged from 2 to 2,600 with the highest number detected in August 2017 (Fig 5C).

From the Coonawarra, analyses of the 865 spore tape samples collected from January 2017 to December 2021, revealed 351 (41%) of the samples tested positive to Diatrypaceous spores (Fig 3B; S3 Table). The highest spore detection was recorded in 2021 (n = 103) and the lowest detection in 2018 (n = 46). The average number of spores detected in all 2-day samples for each month ranged from 2 to 3645 with the highest number detected in June 2020 (Fig 5D).

From McLaren Vale, analyses of the 836 spore tape samples collected from April 2017 to December 2021, revealed 222 (27%) of the samples tested positive to Diatrypaceous spores (Fig 3B; S3 Table). The highest spore detection was recorded in 2020 and 2021 (n = 53 each) and the lowest in 2019 (n = 36). The average number of spores detected in 2-day samples for each month ranged from 4–1600 with the highest numbers detected in August 2017 and January 2020 at 1400 and 1600 spores, respectively (Fig 5E).

From Tumbarumba, NSW, analyses of the 771 samples collected from June 2017 to December 2021, revealed 300 (39%) of the samples tested positive to Diatrypaceous spores (Fig 3B; S3 Table). The highest spore detection was recorded in 2021 (n = 99) and the lowest in 2017 (n = 34). The average number of spores detected in all 2-day samples for each month ranged from 14 to 1800 with seven months having an average of more 1,400 spores with the highest July 2020 and August 2021. In 2020, the spore trap malfunctioned on three occasions resulting in reduced number of samples collected during the season (Fig 5F).

### High resolution melt analysis (HRMA) of spore trap DNA

**Fig 6:**
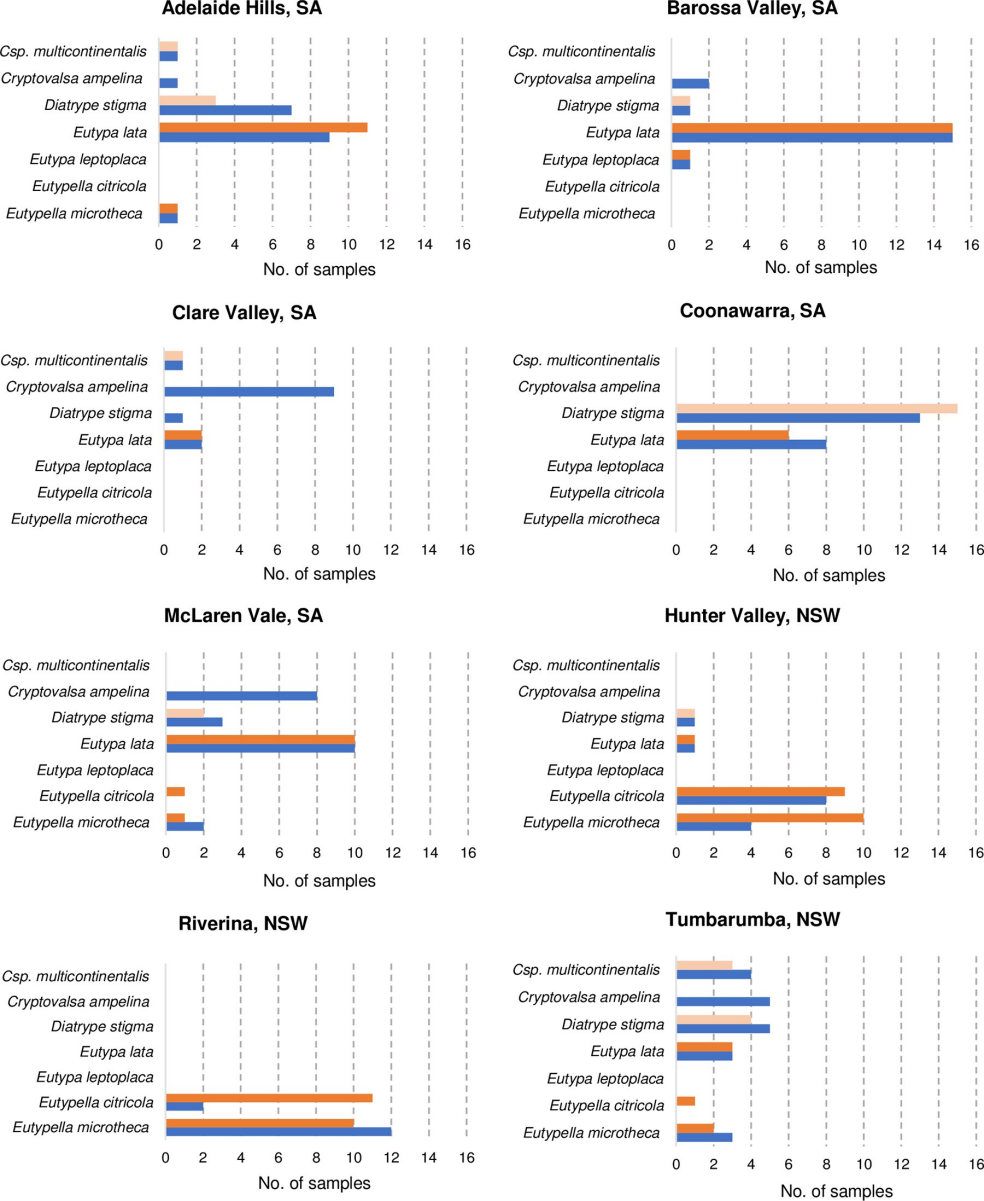
Species diversity of Diatrypaceous airborne spores from different wine growing regions in Australia. Airborne spores were collected using a Burkard volumetric spore trap and DNA extracted from 2-day spore tape sections were identified by High Resolution Melting Analysis (HRMA, ■ ■) and DNA sequencing (■) using Diatrypaceous multi-target primers DITS-1F and DITS-1R that can amplify 305–350 bp of the ribosomal DNA ITS region. Bars in light orange (■) are the DNA samples with similar HRMA curves as *Eutypa lata* and identified as variants. *Csp*. is the abbreviation for the genus *Cryptosphaeria*.

Of the 212 spore tape DNA samples analysed by HRMA, 126 samples were amplified and generated clear and distinguishable HRMA melting profiles. The difference graph which converted *Eu*. *citricola* as a horizontal line revealed clear distinct profiles and the HRMA was able to identify the 48 samples as *E*. *lata*, 24 samples as *Eu*. *microtheca*, 22 samples as *Eu*. *citricola*, one sample as *E*. *leptoplaca* (S4 Fig) while 31 samples were assigned as variants. Subsequent DNA sequencing of the same samples confirmed the identity of the samples, indicating HRMA can be used for rapid identification of Diatrypaceous DNA in mixed samples. However, several samples from the Hunter Valley and Riverina, NSW identified as *Eu*. *citricola* (n = 10) and *Eu*. *microtheca* (n = 6) by HRMA were not confirmed by DNA sequencing due to poor or mixed sequencing reads (Fig 6).

For the 31 samples assigned as variants by HRMA, visual examinations of the difference graphs with *E*. *lata* as core genotype revealed the variants shared similar profiles with *E*. *lata* but were significantly distinct from other species genotypes (S5A Fig). DNA sequencing identified the 32 variants as *Diatrype stigma* (n = 26) and *Cryptosphaeria multicontinentalis* (n = 5; Fig 6), two species not previously reported in Australian vineyards [18]. Alignment and phylogenetic analysis of representative DNA sequences amplified by the primers DITS-1F and the DITS-1R with reference sequences from the GenBank further confirmed the identity of these variants as *Cry*. *multicontinentalis* and *D*. *stigma* (S5A Fig). Furthermore, the 25 samples (out of 86) that were not amplified by HRMA were identified as *C*. *ampelina* by DNA sequencing (Fig 6). The lack of detection of *C*. *ampelina* by HRMA was mainly due to high sequence dissimilarities with the reverse primer DITS-1R.

### Species diversity

Four to five Diatrypaceous species were detected in all regions except for Coonawarra and Riverina where only two species were detected (Fig 6). *Eutypa lata* was the most prevalent species being detected in seven out of eight regions, with highest incidence in the Barossa Valley (n = 15), Adelaide Hills (n = 11), McLaren Vale (n = 10), and Coonawarra (n = 8). One sample was positive to *E*. *lata* in the Hunter Valley, NSW despite *E*. *lata* not being reported in the region in previous studies [18–20]. A newly reported species, *Diatrype stigma* was also prevalent, being detected in seven regions, with highest incidence in Coonawarra (n = 15) and Adelaide Hills (n = 7). *Cryptovalsa ampelina* was detected in five out eight regions with highest incidence in Clare Valley (n = 9) and McLaren Vale (n = 8). *Eutypella microtheca* was detected in five regions with highest incidence in the Riverina (n = 12) and the Hunter Valley (n = 10). *Eutypella citricola* was detected in four regions with the highest incidence in the Riverina (n = 11) and the Hunter Valley (n = 9). *Cryptosphaeria multicontinentalis* was also reported for the first time in three regions with highest detection in Tumbarumba (n = 4). One sample from the Barossa Valley tested positive to *E*. *leptoplaca*.

## Discussion

The diversity and abundance of Diatrypaceous airborne spores from different wine growing regions in Australia were investigated using multi-faceted DNA-based molecular tools. *Eutypa lata* and other Diatrypaceous species are fungal pathogens causing ED in grapevines [2, 15, 17–19]. These pathogens produce airborne ascospores that primarily infect pruning or other wounds and can lead to dieback and cankers in grapevines. This study developed molecular tools for the detection, quantification and identification of the prevalent Diatrypaceous pathogens associated with ED in Australian vineyards. Since vineyard surveys in Australia showed multiple Diatrypaceous fungi were associated with ED [18, 19], the molecular tools in this study were designed to detect multiple Diatrypaceous species, also known to be present in Australian vineyards from spore-trap samples. The multiple-target molecular tools are a more cost effective and less laborious method of detection, quantification and identification of Diatrypaceous airborne inoculum compared to species-specific or multiplex molecular assays.

The qPCR protocol using multi-species primers DIA-17F and DIA-122R developed for this study was able to distinguish between the target species (eight Diatrypaceous species) and other fungal DNA. Specificity tests demonstrated that this primer pair was able to amplify the corresponding Diatrypaceous species, while no PCR products were observed with the DNA of the non-target species tested, including other grapevine fungal pathogens. The qPCR primers developed in this study were highly sensitive since the primers target the ITS region which occurs in multiple copies in a genome, thereby increasing its detection sensitivity. However, the number of rRNA repeats for *E*. *lata* and other Diatrypaceous species is currently unknown, therefore, calculating the number of spores based on the copies of rRNA is difficult. By plotting the different concentrations of gDNA of four representative Diatrypaceous species, with synthetic copies of the rRNA gene (Diat-5S gBlocks) as qPCR standards, the number of copies of the rRNA genes was estimated to be ~110 copies per spore based on the reported size of the *E*. *lata* haploid genome which is ~54 mbp (or 54 fg) [52]. The qPCR protocol developed in this study was able to detect up to 20 copies of the Diat-5S gBlocks or 20 fg of gDNA, theoretically less than one spore. The limit of detection of 20 fg is more sensitive than the qPCR developed for *Ph*. *chlamydospora* with a detection limit of 108 fg of gDNA using the multi-copy rRNA gene as the target [56].

In this study, a second primer pair DITS-1F and DITS-1R designed to amplify 305–350 bp of the ITS region of Diatrypaceous fungi was shown to be highly suitable for the detection of DNA from six and seven Diatrypaceous species, using conventional PCR and nested PCR, respectively. This primer pair was useful for screening the spore tape samples for the presence or absence of Diatrypaceous DNA by nested-PCR in tandem with the DIA-17F and DIA-122R qPCR primers. These primers were also found to be suitable with HRMA for rapid identification of at least six Diatrypaceous species commonly reported in Australian vineyards in mixed samples.

Previous studies on spore dispersal patterns of ED [3, 4, 24, 27, 57–60] relied primarily on conventional techniques such as microscopy and culturing on artificial media that are extremely time consuming, less accurate and less sensitive than molecular tools for detecting pathogens from environmental samples. In recent years, PCR-based techniques have been developed for the detection and diagnostics of *E*. *lata*, due to their accuracy and sensitivity compared to conventional plant pathology techniques. The development of qPCR assays capable of detecting *E*. *lata* from grapevine wood [33, 34] and spore tape samples [35] have detected this pathogen in different environmental samples. However, the above-mentioned qPCR assays are only specific to *E*. *lata*. The qPCR protocols using multi-species primers developed in this study are rapid and more cost effective for accurate detection and quantification of Diatrypaceous DNA from a single sample. To our knowledge, these are the first qPCR methods developed to target multiple Diatrypaceous species from environmental samples (in this case from spore tape samples). A similar multi-target qPCR assays was developed for detecting multiple Botryosphaeriaceae airborne spores in Australian vineyards [31].

The qPCR primers developed in this study were designed to amplify multiple Diatrypaceous species making them cost-effective. However, they are not suitable for discriminating multiple species from environmental samples using conventional or qPCR. Identification of the different species from individual spore tapes will assist in determining the abundance of each species in each region. Multiplex qPCR is a popular method for detecting multiple species in a single sample. A multiplex qPCR assay was developed to quantify *Ph*. *chlamydospora* and *P*. *aleophilum* from infected grapevine wood samples [61] but to our knowledge a published multiplex qPCR for Diatrypaceous species is not available to date. Furthermore, the primers and probes for multiplex PCR are generally difficult to design and optimise compared with singleplex qPCR. A conventional PCR-based method coupled with single-stranded conformation polymorphism (SSCP) analysis was developed to discriminate mixed Botryosphaeriaceae species amplified from environmental samples [62]. DNA macroarrays were also developed to detect several species of pathogens causing young vine decline from single nursery planting materials, including Petri/esca disease and black foot pathogens [63]. While these methods are highly sensitive and effective in detecting multiple species in one sample, these molecular tools require multiple post-PCR steps that are generally complex and time consuming. Thus, a simple and rapid PCR-based method for simultaneous identification of Diatrypaceous species could be a useful tool for Diatrypaceous spore surveillance studies.

In this study, a PCR-based HRMA method was also developed that can rapidly discriminate at least six Diatrypaceous species reported in Australian vineyards. Furthermore, the complex melt profiles of *D*. *vulgaris*, *E*. *lata* and *E*. *leptoplaca* yielded two distinct peaks which were in contrast with the single melt peaks for *C*. *rabenhorstii*, *Eu*. *citricola* and *Eu*. *microtheca*. The qPCR using intercalating dye generally uses the post-PCR melt curve analysis to assess the specificity of amplification with the assumption that a single amplicon will produce single peak while two peaks is indicative of two separate amplicons or non-specific amplifications [39, 64]. However, this study revealed, *D*. *vulgaris*, *E*. *lata* and *E*. *leptoplaca* with larger PCR products yielded complex and double melt peak profiles by HRMA despite gel electrophoresis confirming the presence of a single PCR product. These results conform with previous studies showing that longer DNA fragments can give rise to complex melt profiles and that one amplicon can exhibit two or more peaks [39, 65]. Thus, the complex melting curves reported in this study are most likely due to the larger size of PCR products amplified by the DITS-1F and DITS-1R primers for these three species (350–351 bp), which can be useful for rapid visual identification of these species by HRMA.

In this study, the HRMA has been shown to be highly effective in distinguishing the three most prevalent species (*E*. *lata*, *Eu*. *citricola* and *Eu*. *microtheca*) from spore tape samples and correlated with the DNA sequencing results. The other reference genotypes (*D*. *vulgaris* and *C*. *rabenhorstii*) were not detected in any of the spore tape samples tested, indicating that these species were not common in the spore surveillance sites selected in this study. This contrasts with the vineyard survey results where a small number of *D*. *vulgaris* were isolated from *V*. *vinifera* in Tumbarumba, NSW and *Fraxinus angustiolia* in the Hunter Valley, while two *C*. *rabenhorstii* isolates were recovered from *V*. *vinifera* in Western Australia [18].

The DNA of *C*. *ampelina* was successfully amplified by nested PCR and qPCR allowing detection of this species from spore tape samples. These results were subsequently confirmed by DNA sequencing. However, this species was not amplified by the HRMA that employs a highly stringent DNA polymerase [53], since this species has a two bases dissimilarity with the reverse primer DITS-1R. Since *C*. *ampelina* was shown to be a prevalent species by DNA sequencing, detection of this species using other molecular methods is essential to ensure this species is not excluded when determining species prevalence. Knowledge of the prevalence of *C*. *ampelina* in vineyards will assist in understanding the epidemiology of this species and its role in symptoms development in the field.

The HRMA further detected two new Diatrypaceous species from the spore surveillance samples. These two new species were assigned as variants and were distinguishable from *E*. *lata* based on the second peaks of their melt curve profiles. The subsequent DNA sequencing identified these variants as *D*. *stigma* and *Cry*. *multicontinentalis*. Expanding the reference genotypes for HRMA with the inclusion of DNA samples for *D*. *stigma* and *Cry*. *multicontinentalis* will allow rapid identification of these two species in future spore surveillance studies in Australia.

Unlike PCR-SSCP [62] and DNA macroarrays [63], that can detect and identify multiple species in a single sample, HRMA can only detect the most abundant pathogen DNA present in a sample. Since the melting temperature of the PCR product can also be affected by the target DNA concentration [38, 39], all spore tape samples used for HRMA in this study were pre-amplified to ensure high fluorescence signal and reproducible performance. The HRMA is a rapid and cost-effective method for simultaneous identification of prevalent Diatrypaceous pathogens in environmental samples. It has been shown as a rapid and accurate method for genotyping pathogens such as *B*. *cinerea* [37] and *V*. *inaequalis* [38]. This approach was also used as a rapid method for simultaneous identification of fungal pathogens such as black aspergilli from grapes [41] and *Alternaria* species causing citrus brown spot [42].

The research presented here was also the first attempt to comprehensively investigate the spore release patterns of Diatrypaceous pathogens in eight wine regions of Australia using multi-faceted molecular tools. Prior to this study, information on spore dispersal of *E*. *lata* in Australia was based on a study in apricot orchards conducted in the 1960s [57]. Analysis of ~7000 spore tape samples collected over 8 years showed Diatrypaceous spores were released at different times of the year, but the seasonal release and number of spores differed between regions, months and years. In the Barossa Valley, the number of ED spores were generally lower (4 to 5-fold lower) compared to other regions such as Coonawarra, McLaren Vale and Tumbarumba. The spore trap at the Barossa Valley was placed ~ 20 m away from the vineyard rows while the spore traps for the other regions were located at the end of a row. Thus, the largest distance between the trap and vine for the Barossa Valley may explain the lower spore numbers detected. The seasonal release of Diatrypaceous spores also varied, with a higher percentage of spores detected in winter and spring.

This study showed rainfall as a primary factor that triggers the release of Diatrypaceous spores and is in general agreement with past studies in vineyards in California [3, 17], Michigan [4] and New York [66] in the United States. At all spore trapping sites, Diatrypaceous spores were generally detected during or immediately after the occurrence of rain. On some occasions, spores were also trapped several days after a rain event. The detection of spores in the absence of rain suggests other climatic factors such as relative humidity and dew point may also play a role in the release of these spores. Further investigation is underway using computer modelling of the spore detection data to elucidate the climatic factors that contribute to the release of spores for these species as well as for species of the Botryosphaeriaceae.

HRMA coupled with DNA sequencing of spore tape samples positive to Diatrypaceous spores identified seven Diatrypaceous species, with *E*. *lata* being present in seven out of eight regions, and the most prevalent species in three regions of SA. However, *C*. *ampelina* and a newly reported species *D*. *stigma* were more prevalent than *E*. *lata* in two other SA regions. *Eu*. *microtheca* and *Eu*. *citricola* were most prevalent in two NSW regions while barely detected in SA. The Australian survey [18] showed that *Eu*. *microtheca* was the most prevalent species isolated from the Hunter Valley. The present study also identified one sample from the Hunter Valley as positive to *E*. *lata*, although this species was not reported in this region in previous studies [18–20]. Thus, it is important to monitor for ED foliar symptoms to further confirm the presence and prevalence of *E*. *lata* in the Hunter Valley. These findings further suggest climatic conditions may play a role in the prevalence and distribution of Diatrypaceous species since spore surveillance studies were carried out in wine regions with highly diverse climatic conditions [67]. However, a correlation between climate and species prevalence requires further investigation.

This study represents the first report of *D*. *stigma* and *Csp*. *multicontinentalis* in Australian vineyards. One isolate each of *Diatrype* sp. and *Cryptosphaeria* sp. have previously been isolated from grapevines in Tumbarumba, NSW and poplar in Khancoban, NSW, respectively but were not identified to species level [18]. It is likely these species have been present in Australia without identification for some time. *Diatrype stigma* has been reported as a pathogen of grapevine in California [17], while *Csy*. *multicontenitalis* has been reported as a pathogen of poplars in South Africa [68]. Since these two new species were only detected by PCR and not by fungal isolation from infected wood, their association with the dieback symptoms observed in vineyards requires further investigation.

This is the first comprehensive investigation on the diversity and abundance of Diatrypaceous species in Australian vineyards using multi-faceted molecular tools. Spore surveillance studies on multiple Diatrypaceous species in vineyards are limited only to studies carried out in British Columbia, Canada [23] and in Southern California [24]. Pruning of wine grapes in Australia normally takes place during winter (June–August) when vines are dormant, which usually coincides with the highest rainfall periods. Since the wine regions in Australia cover a wide area with highly diverse climates, the comprehensive spore trapping in major wine regions in this study provided beneficial information on the spore release patterns of Diatrypaceous species in different regions. The molecular tools developed in this study fast-tracked the analyses of the spore tape samples and allowed quantification and identification of Diatrypaceous spores from vineyards. The data generated from this study will assist in determining the climatic conditions required for spore release by Diatrypaceous pathogens and inoculum dispersal throughout the growing season, and, in particular, during the winter pruning season and spring shoot thinning activities in different climatic regions of Australia.

## Supporting information

S1 Fig DNA sequences for the Diat-5S gBlocks^®^ (Integrated DNA Technologies, USA) gene fragment used for the construction of the quantitative PCR standard curve. The Diat-5S gBlocks^®^ was designed based on the *Eutypa lata* ITS sequences and shared sequence homology with DIA-17F and DIA-122R primers. The primer binding sites are shown in red bold letters.
(PDF)

S2 Fig Australian map showing the geographic locations of the eight wine growing regions (green) where the spore surveillance studies were conducted. The name and location of the regions are indicated by numbers. The map was created using ESRI ArcGIS Pro 3.0.2. The base map is sourced from the **Australian Bureau of Statistics** https://www.abs.gov.au/statistics/standards/australian-statistical-geography-standard-asgs-edition-3/jul2021-jun2026/access-and-downloads/digital-boundary-files and **Wine GI Regions** https://wineaustralia-opendata-wineaustralia.hub.arcgis.com/maps/ede7ffb0e73b4504a5ed613965b11e0f/about, 13 February 2023.
(PDF)

S3 Fig Representative 1% agarose gel of PCR products amplified by multi-species primers DITS-1F and DITS-1R using genomic DNA of Diatrypaceae species. (A) Lane 1, HyperLadder™ 50 bp (Meridian Bioscience, USA); lanes 2–10, *Eutypa lata*; lanes 11–15, *Cryptovalsa ampelina*, lanes 16–20, *E*. *leptoplaca*; (B) Lane 1, HyperLadder™ 50 bp (Meridian Bioscience, USA); lanes 2–6, *Eutypella citricola*, lanes 7–8, *Diatrypella vulgaris*, lanes 9–10, *Eu*. *microtheca*, lane 11. *C*. *rabenhorstii*; lanes 12–19 non-Botryosphaeriaceae species (non-target species) and lane 20, non-template control. The number of the far left denote the molecular weight of the 50 bp ladder.
(PDF)

S4 FigHigh resolution melting analysis (HRMA) of representative Diatrypaceous DNA from spore trap samples collected from different winegrowing regions in Australia.The difference graph grouped the unknown spore trap samples into four clusters. Thin solid lines represent the unknown spore trap samples. The dotted lines are the reference genotypes: *Eutypa lata* (•••••), *E*. *leptoplaca* (*●●●●●*), *Eutypella citricola* (••••• horizontal line) and *Eu*. *microtheca* (•••••).(PDF)

S5 Fig(A) Difference graphs produced by *Eutypella citricola* and the two representative spore tape samples that were assigned as variants by High Resolution Melting Analysis (HRMA) with *Eutypa lata* assigned as the core genotype and converted to a horizontal line. (B) Neighbour joining tree based on the DNA sequences for spore tape samples from different regions identified as variants 1 (●) and variants 2 (●) by HRMA using the DITS-1F and DITS-1R primers. The DNA sequences of E. lata, Cryptosphaeria multicontinentalis, Diatrype stigma and Australian isolates of Cryptosphaeria sp., D. brunneospora, Diatrype sp. retrieved from Genbank and used as references are indicated by their accession numbers. The reference sequence of Cryptovalsa ampelina from the Genbank was used as an outgroup. The bootstrap values are indicated on the nodes based on 1,000 bootstrap replicates.(PDF)

S1 Table Australian wine growing regions and the corresponding cultivars, planting dates and climatic conditions where spore surveillance studies were conducted. (PDF)

S2 Table Total number of 2-day spore tape samples collected from the four wine growing regions in 2014–2016 and the percentage of samples that tested positive to Diatrypaceous spores from. (PDF)

S3 Table Total number of 2-day spore tape samples collected from the six wine growing regions in 2017–2021 and the percentage of samples that tested positive to Diatrypaceous spores. (PDF)
