# Supplementary material for: Molecular detection and identification of Diatrypaceous airborne spores in Australian vineyards revealed high species diversity between regions
Source: PLoS One. 2023 Jun 2;18(6):e0286738. doi: 10.1371/journal.pone.0286738 (PMC10237649; doi:10.1371/journal.pone.0286738)
Supplement: S1 Fig — The Diat-5S gBlocks® was designed based on the Eutypa lata ITS sequences and shared sequence homology with DIA-17F and DIA-122R primers. The primer binding sites are shown in red bold letters. (PDF) [file pone.0286738.s001.pdf]

GTCTCCGTTGGTGAACCAGCGGAGGGATCATTACAGAGTTACCTAACTCCAAA

CCCATGTGAACTTACCTATGTTGCCTCGGCGGGGAAGCCTACCCGGTACCTAC

CCTGTAGCTACCCGGGAGCGAGCTACCCTGTAGCCCGCTGCAGGCCTACCCGC

CGGTGGACACTTAAACTCTTGTTTTTAGTGATTATCTGAGTGTTTATACTTA

Diat-17F

ATAAGTTCCAACTTTCAACAAC**GGATCTCTTGGTTCTGGCAT**CGATGAAGAAC

GCAGCGAAATGCGATAAGTAATGTGAATTGCAGAATTCAGTGAATCATCGAAT

Diat-122R

CTTTGAACGCACATTGCGCCC**ATTAGTATTCTAGTGGGCAT**GCCTGTTCGAGC

GTCATTTCGACCTTCAAGCCCTAGCTGCTTGGTGTTGGGAGCCTATCTCCGGA

TAGCTCCTCAAAAGCATTGGCGGAGTCGCGGTGACC

S1 Fig. DNA sequences for the Diat-5S gBlocks® (Integrated DNA Technologies, USA) gene fragment used for the construction of the quantitative PCR standard curve. The Diat-5S gBlocks® was designed based on the *Eutypa lata* ITS sequences and shared sequence homology with DIA-17F and DIA-122R primers. The primer binding sites are shown in red bold letters.
