# Supplementary material for: Molecular detection and identification of Diatrypaceous airborne spores in Australian vineyards revealed high species diversity between regions
Source: PLoS One. 2023 Jun 2;18(6):e0286738. doi: 10.1371/journal.pone.0286738 (PMC10237649; doi:10.1371/journal.pone.0286738)
Supplement: S2 Fig — The name and location of the regions are indicated by numbers. The map was created using ESRI ArcGIS Pro 3.0.2. The base map is sourced from the Australian Bureau of Statistics https://www.abs.gov.au/statistics/standards/australian-statistical-geography-standard-asgs-edition-3/jul2021-jun2026/access-and-downloads/digital-boundary-files and Wine GI Regions https://wineaustralia-opendata-wineaustralia.hub.arcgis.com/maps/ede7ffb0e73b4504a5ed613965b11e0f/about, 13 February 2023. (PDF) [file pone.0286738.s002.pdf]

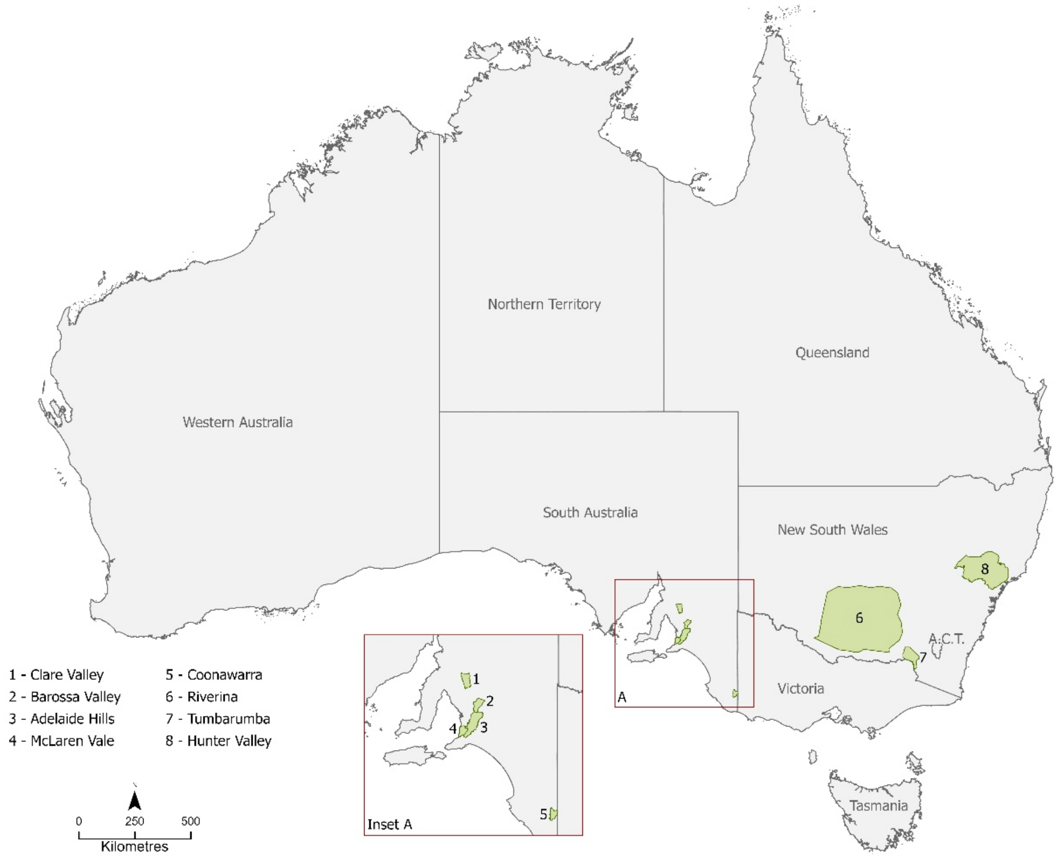

S2 Fig. Australian map showing the geographic locations of the eight wine growing regions (green) where the spore surveillance studies were conducted. The name and location of the regions are indicated by numbers. The map was created using ESRI ArcGIS Pro 3.0.2. The base map is sourced from the **Australian Bureau of Statistics** https://www.abs.gov.au/statistics/standards/australian-statistical-geography-standard-asgs-edition-3/jul2021-jun2026/access-and-downloads/digital-boundary-files and **Wine GI Regions** https://wineaustralia-opendata-wineaustralia.hub.arcgis.com/maps/ede7ffb0e73b4504a5ed613965b11e0f/about, 13 February 2023.
