# Supplementary material for: Molecular detection and identification of Diatrypaceous airborne spores in Australian vineyards revealed high species diversity between regions
Source: PLoS One. 2023 Jun 2;18(6):e0286738. doi: 10.1371/journal.pone.0286738 (PMC10237649; doi:10.1371/journal.pone.0286738)
Supplement: S3 Fig — (A) Lane 1, HyperLadder™ 50 bp (Meridian Bioscience, USA); lanes 2–10, Eutypa lata; lanes 11–15, Cryptovalsa ampelina, lanes 16–20, E. leptoplaca; (B) Lane 1, HyperLadder™ 50 bp (Meridian Bioscience, USA); lanes 2–6, Eutypella citricola, lanes 7–8, Diatrypella vulgaris, lanes 9–10, Eu. microtheca, lane 11. C. rabenhorstii; lanes 12–19 non-Botryosphaeriaceae species (non-target species) and lane 20, non-template control. The number of the far left denote the molecular weight of the 50 bp ladder. (PDF) [file pone.0286738.s003.pdf]

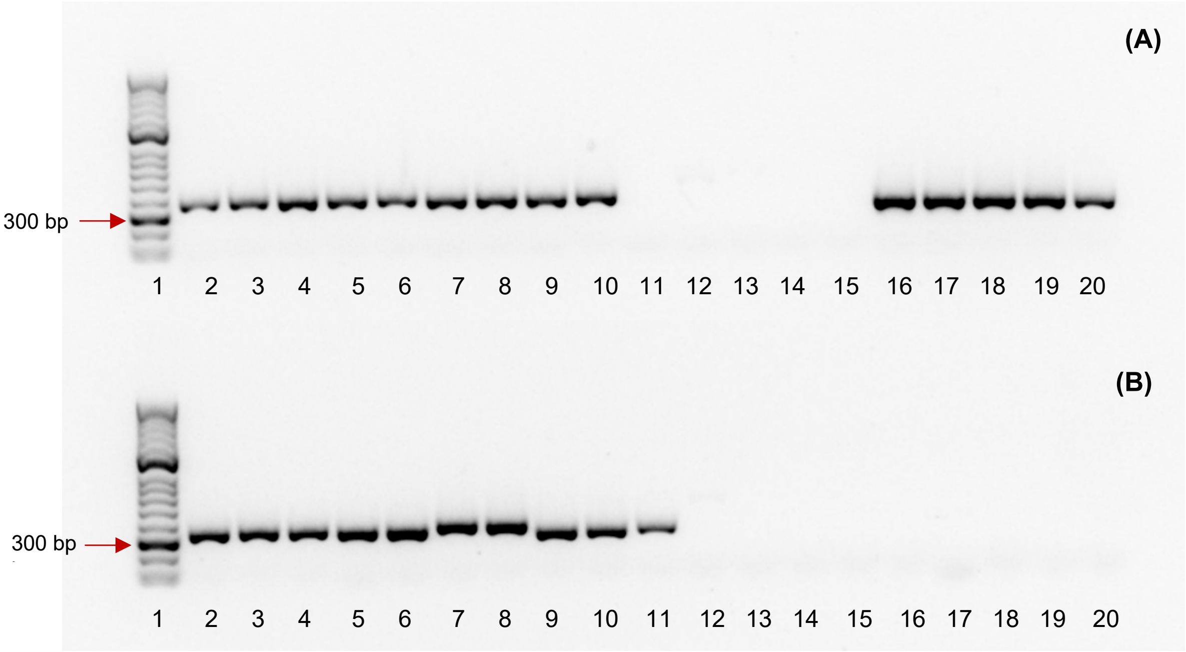

S3 Fig. Representative 1% agarose gel of PCR products amplified by multi-species primers DITS-1F and DITS-1R using genomic DNA of Diatrypaceae species. (A) Lane 1, HyperLadder™ 50 bp (Meridian Bioscience, USA); lanes 2-10, *Eutypa lata*; lanes 11-15, *Cryptovalsa ampelina*, lanes 16-20, *E. leptoplaca*; (B) Lane 1, HyperLadder™ 50 bp (Meridian Bioscience, USA); lanes 2-6, *Eutypella citricola*, lanes 7-8, *Diatrypella vulgaris*, lanes 9-10, *Eu. microtheca*, lane 11. *C. rabenhorstii;* lanes 12-19 non-Botryosphaeriaceae species (non-target species) and lane 20, non-template control. The numbers on the far left for each gel denote the molecular weight of the 50 bp ladder.
