# Supplementary material for: Molecular detection and identification of Diatrypaceous airborne spores in Australian vineyards revealed high species diversity between regions
Source: PLoS One. 2023 Jun 2;18(6):e0286738. doi: 10.1371/journal.pone.0286738 (PMC10237649; doi:10.1371/journal.pone.0286738)
Supplement: S4 Fig — The difference graph grouped the unknown spore trap samples into four clusters. Thin solid lines represent the unknown spore trap samples. The dotted lines are the reference genotypes: Eutypa lata (•••••), E. leptoplaca (●●●●●), Eutypella citricola (••••• horizontal line) and Eu. microtheca (•••••). (PDF) [file pone.0286738.s004.pdf]

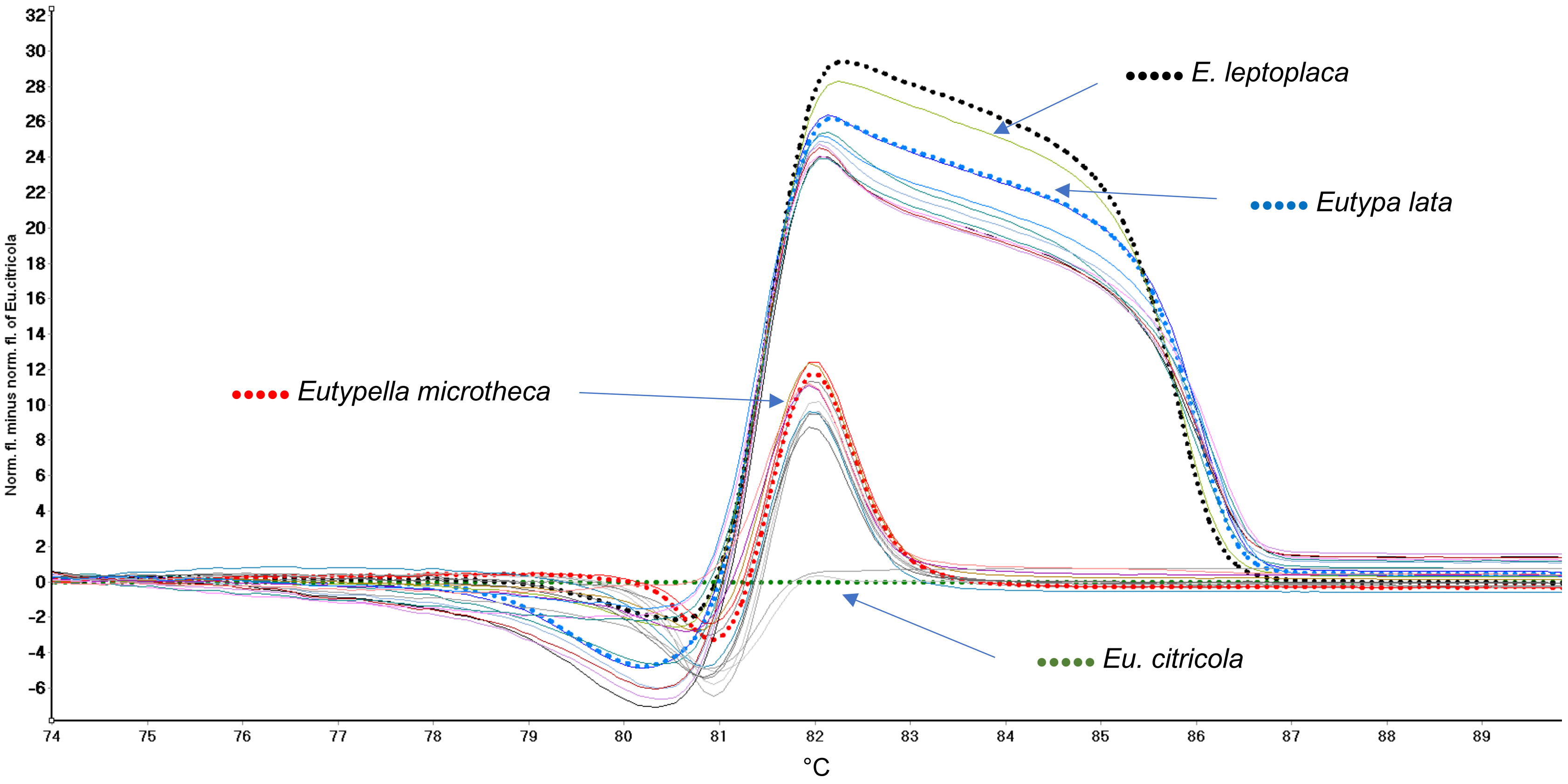

S4 Fig. High resolution melting analysis (HRMA) of representative Diatrypaceous DNA from spore trap samples collected from different winegrowing regions in Australia. The difference graph grouped the unknown spore trap samples into four clusters. Thin solid lines represent the unknown spore trap samples. The dotted lines are the reference genotypes: *Eutypa lata* (●●●●●), *E. leptoplaca* (●●●●●), *Eutypella citricola* (●●●●● horizontal line) and *Eu. microtheca* (●●●●●).
