# Supplementary material for: Molecular detection and identification of Diatrypaceous airborne spores in Australian vineyards revealed high species diversity between regions
Source: PLoS One. 2023 Jun 2;18(6):e0286738. doi: 10.1371/journal.pone.0286738 (PMC10237649; doi:10.1371/journal.pone.0286738)
Supplement: S5 Fig — (A) Difference graphs produced by Eutypella citricola and the two representative spore tape samples that were assigned as variants by High Resolution Melting Analysis (HRMA) with Eutypa lata assigned as the core genotype and converted to a horizontal line. (B) Neighbour joining tree based on the DNA sequences for spore tape samples from different regions identified as variants 1 (●) and variants 2 (●) by HRMA using the DITS-1F and DITS-1R primers. The DNA sequences of E. lata, Cryptosphaeria multicontinentalis, Diatrype stigma and Australian isolates of Cryptosphaeria sp., D. brunneospora, Diatrype sp. retrieved from Genbank and used as references are indicated by their accession numbers. The reference sequence of Cryptovalsa ampelina from the Genbank was used as an outgroup. The bootstrap values are indicated on the nodes based on 1,000 bootstrap replicates. (PDF) [file pone.0286738.s005.pdf]

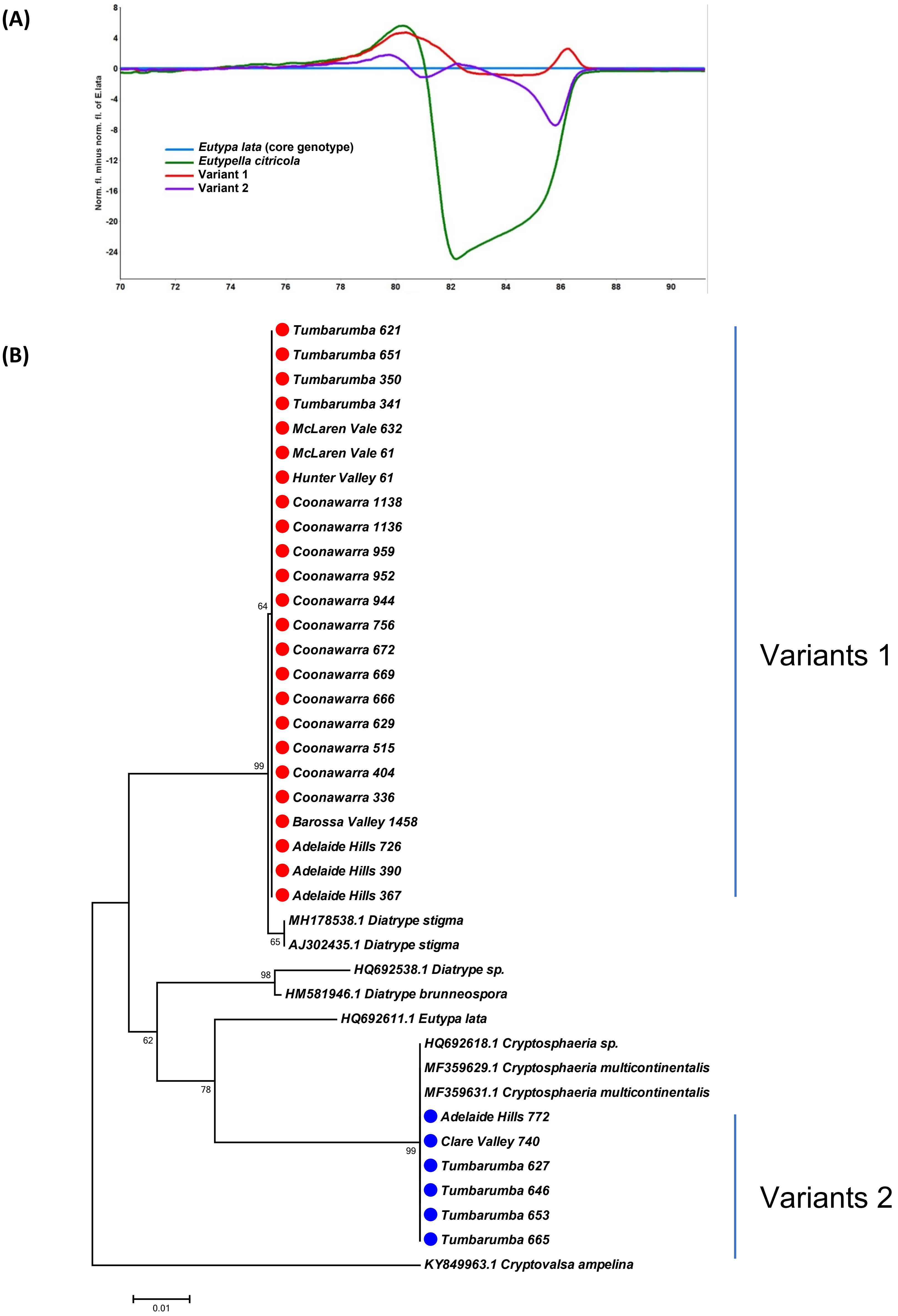

S5 Fig. (A) Difference graphs produced by *Eutypella citricola* and the two representative spore tape samples that were assigned as variants by High Resolution Melting Analysis (HRMA) with *Eutypa lata* assigned as the core genotype and converted to a horizontal line. (B) Neighbour joining tree based on the DNA sequences for spore tape samples from different regions identified as variants 1 (●) and variants 2 (●) by HRMA using the DITS-1F and DITS-1R primers. The DNA sequences of *E. lata, Cryptosphaeria multicontinentalis*, *Diatrype stigma* and Australian isolates of *Cryptosphaeria sp., D. brunneospora, Diatrype sp.* retrieved from Genbank and used as references are indicated by their accession numbers. The reference sequence of *Cryptovalsa ampelina* from the Genbank was used as an outgroup. The bootstrap values are indicated on the nodes based on 1,000 bootstrap replicates.
