# Supplementary material for: Molecular detection and identification of Diatrypaceous airborne spores in Australian vineyards revealed high species diversity between regions
Source: PLoS One. 2023 Jun 2;18(6):e0286738. doi: 10.1371/journal.pone.0286738 (PMC10237649; doi:10.1371/journal.pone.0286738)
Supplement: S1 Table — (PDF) [file pone.0286738.s006.pdf]

S1 Table. Australian wine growing regions and the corresponding cultivars, planting dates and climatic conditions where spore surveillance studies were conducted.

Region	Sampling dates	Cultivar/s	Planted	[a] Climate
**South Australia**				
Adelaide Hills	2017-2021	Sauvignon Blanc, Shiraz	2004, 2007, 1997	Warm-wet
Barossa Valley	2014-2016	Savagnin Blanc, Shiraz	2001, 2008	Warm-dry
	2017-2021	Savagnin Blanc, Shiraz	2001, 2008	Warm-dry
Clare Valley	2017-2021	Shiraz	2006	Warm-dry
Coonawarra	2014-2016	Cabernet Sauvignon	Circa 1984-88	Cool
	2017-2021	Cabernet Sauvignon	Circa 1984-88	Cool
McLaren Vale	2017-2021	Cabernet Sauvignon	1962	Warm-dry
**New South Wales**				
Hunter Valley	2014-2016	Semillon	1994	Hot-wet
Riverina	2014-2016	Chardonnay	1999	Hot-dry
Tumbarumba	2017-2021	Pinot Noir	1995	Cool

[a] Dry, PR and Coombe, BG (editors), (2008) Viticulture, Vol 1 – Resources, 2nd ed., Winetitles Pty Ltd, Broadview, South Australia.
