# Supplementary material for: Molecular detection and identification of Diatrypaceous airborne spores in Australian vineyards revealed high species diversity between regions
Source: PLoS One. 2023 Jun 2;18(6):e0286738. doi: 10.1371/journal.pone.0286738 (PMC10237649; doi:10.1371/journal.pone.0286738)
Supplement: S2 Table — (PDF) [file pone.0286738.s007.pdf]

S2 Table. Total number of 2-day spore tape samples collected from the four wine growing regions in 2014-2016 and the percentage of samples that tested positive to Diatrypaceous spores.

[a] Region	Sampling dates	Total no. of 2-day samples	2-day samples positive to spores (%)	Calculated no. of spores
Barossa Valley, SA	Jan 2014-Dec 2016	537	126 (23%)	30 - 3,483
Coonawarra, SA	Jan 2014-Dec 2016	426	100 (23%)	30 - 10,608
Riverina, NSW	Mar 2014-Nov 2016	408	102 (25%)	30 - 12,450
Hunter Valley, NSW	May 2014-Oct 2016	425	145 (34%)	30 - 30,162
TOTAL		1,796	473 (26%)	

[a] SA – South Australia; NSW – New South Wales
