# Supplementary material for: Molecular detection and identification of Diatrypaceous airborne spores in Australian vineyards revealed high species diversity between regions
Source: PLoS One. 2023 Jun 2;18(6):e0286738. doi: 10.1371/journal.pone.0286738 (PMC10237649; doi:10.1371/journal.pone.0286738)
Supplement: S3 Table — (PDF) [file pone.0286738.s008.pdf]

S3 Table. Total number of 2-day spore tape samples collected from the six wine growing regions in 2017-2021 and the percentage of samples that tested positive to Diatrypaceous spores.

[a] Region	Sampling dates	Total no. of 2-day samples	2-day samples positive to spores (%)	Calculated no. of spores
Adelaide Hills, SA	Apr 2017 – Dec 2021	868	322 (37%)	30-14,542
Barossa Valley, SA	Jan 2017 – Nov 2021	912	222 (24%)	30-6,500
Clare Valley, SA	Mar 2017 – Nov 2021	837	192 (23%)	30-7455
Coonawarra, SA	Jan 2017 – Nov 2021	865	351 (41%)	30-25,298
McLaren Vale, SA	Apr 2017 – Dec 2021	836	222 (27%)	30-17,599
Tumbarumba, NSW	Jun 2017 – Dec 2021	771	300 (39%)	30-25,478
TOTAL		5071	1652 (33%)	

[a] SA – South Australia; NSW – New South Wales
